# Prevalence of post-traumatic stress disorder in healthcare workers before and during COVID-19: a systematic review and meta-analysis

**DOI:** 10.3389/fpubh.2026.1735552

**Published:** 2026-03-06

**Authors:** Claire Frodsham, Samuel B. Harvey, Daniel Collins, Karen Krakue, Vita Ligaya Dalgaard, Rosie Lipscomb, Matthew Hotopf, Mark Deady, Richard Bryant, Aimee Gayed

**Affiliations:** 1 Black Dog Institute, Faculty of Medicine & Health, University of New South Wales, Sydney, NSW, Australia; 2 Department of Psychology and Behavioural Sciences, School of Business and Social Sciences, Aarhus University, Aarhus, Denmark; 3 Department of Psychological Medicine, King’s College London, Institute of Psychiatry, Psychology and Neuroscience, London, United Kingdom; 4 School of Psychology, University of New South Wales, Sydney, NSW, Australia

**Keywords:** COVID-19, health personnel, meta-analysis, post-traumatic stress discorder, prevalence

## Abstract

The COVID-19 pandemic exacerbated many known risk factors for post-traumatic stress disorder (PTSD) among healthcare workers. This systematic review and meta-analysis examines pooled prevalence estimates of probable PTSD among this cohort prior to COVID-19 compared to during COVID-19 and investigates time trends in prevalence. Systematic multi-database literature searches were conducted to identify studies published between January 2017 and July 2023. Included studies reported the prevalence of probable PTSD, measured by validated screening tools, in clinical healthcare workers. Two reviewers independently conducted study screening, data extraction, and quality assessment. Random-effects meta-analyses were performed to estimate pooled prevalence of probable PTSD among healthcare workers in each time period. Subgroup analyses were carried out for year, profession, quality of study, COVID-19 mortality rates, and income level within the country of study. Screening identified 21 papers comprising 11,838 healthcare workers published in the 3 years preceding the pandemic, and 129 papers reporting on 130,363 healthcare workers during the pandemic. The pooled prevalence estimate of probable PTSD prior to the pandemic was 15.5% (95% CI: 12.3–19.3%) and this significantly increased during the pandemic to 24.8% (95% CI: 22.0–27.8%), peaking early in the pandemic in 2020 before returning to pre-pandemic levels in 2022. During the pandemic, prevalence estimates were significantly higher among nurses and those in countries with high COVID-19 mortality rates, whilst no significant difference was observed between studies conducted in high-income versus low- and middle-income countries. Substantial heterogeneity was observed. The findings of this review suggest that prevalence of PTSD among healthcare workers significantly increased following the COVID-19 outbreak. By the third year of the pandemic, probable PTSD prevalence rates appear to return to pre-pandemic levels, although these levels remain concerningly high. These findings support the call for targeted interventions to protect healthcare worker wellbeing, particularly during healthcare emergencies.

**Systematic review registration:**

https://www.crd.york.ac.uk/PROSPERO/view/CRD42022364955, unique identifier is CRD42022364955

## 1 Introduction

Post-traumatic stress disorder (PTSD) affects approximately 1.1% of the global population annually and can occur as a result of a single traumatic event or cumulative trauma exposure (1). Healthcare workers (HCWs), including physicians, nurses and other patient-facing healthcare personnel, are frequently exposed to potentially traumatic experiences in the workplace, putting them at increased risk of developing PTSD (2). While experiencing or witnessing trauma is the primary cause of PTSD, other factors associated with healthcare work, such as long working hours, high workloads, emotionally demanding work, and moral injury have also been recognized as risk factors for PTSD (3, 4).

Coronavirus disease 2019 (COVID-19) emerged in December 2019 and spread rapidly worldwide before being declared a global pandemic in March 2020 by the World Health Organization. As of April 2025, the World Health Organization had reported over 750 million cases and 7 million COVID-19 deaths globally (5). The rapid increase in demand for healthcare services placed considerable pressure on healthcare systems and HCWs worldwide, with little time to adequately prepare. HCWs faced many unique challenges during the pandemic, including moral injury due to insufficient resources, risk of infection, redeployment to areas outside their specialism, and insufficient access to personal protective equipment (PPE) (6, 7). These factors have all been consistently identified as sources of distress in qualitative research (6, 8) and have been associated with a greater risk of mental health disorders, including PTSD, among HCWs (4, 9–11), leading to a concern about a possible increase in post-traumatic stress symptoms in the wake of the COVID-19 pandemic.

Several systematic reviews and meta-analyses have addressed the prevalence of PTSD in HCWs during the COVID-19 pandemic, reporting estimates between 11 and 38% (11–16). However, these reviews have some key shortcomings, including being conducted in the early stages of the pandemic, and the lack of direct comparisons to the pre-COVID-19 period. As a result, the key question of whether COVID-19 has generated increased levels of PTSD amongst HCWs remains unanswered. To the best of our knowledge, there has yet to be a systematic review and meta-analysis published that assesses pre-pandemic rates of PTSD among HCWs and presents this information with a comparison to PTSD prevalence rates during the pandemic. This systematic review and meta-analysis aims to address this gap by examining the prevalence of PTSD prior to COVID-19 and during each year of the COVID-19 pandemic.

## 2 Methods

This systematic review and meta-analysis was conducted and reported according to Preferred Reporting Items for Systematic reviews and Meta-Analyses (PRISMA) guidelines.

### 2.1 Search strategy

We conducted two independent searches for papers to identify studies (a) published in the 3 years prior to COVID-19 from January 2017 until November 2019, and (b) conducted during the COVID-19 pandemic and published between December 2019 and July 2023. PsycInfo, Embase, Cochrane, PubMed, and ProQuest Coronavirus Research Databases were searched using keywords related to healthcare workers, PTSD, and prevalence. A detailed search strategy and a copy of the search strings that were used are provided in Supplementary Appendix 1.

### 2.2 Study selection

Covidence, a systematic review software, was employed for title and abstract screening, full text screening, data extraction and quality assessment, each of which were conducted independently by two reviewers of CF, KK, DC, RL, VLD, NK and EG. Where required, consensus was reached between the two members through discussion, and the supervising author (AG) was consulted in instances where consensus was not reached.

Inclusion criteria required peer-reviewed, observational studies, with a translation available in English, published between January 2017 and December 2019 for papers prior to COVID-19 or published between December 2019 and July 2023 and reporting on data captured during the pandemic for during COVID-19 papers. Papers must have reported on the prevalence of PTSD in patient-facing, clinical healthcare workers, which were grouped into physicians, nurses, paramedics or “other healthcare workers” for analysis. The “other healthcare workers” category consisted of qualified or trainee health professionals not otherwise classified, including allied health professionals, midwives, student healthcare workers, and healthcare assistants. Studies reporting on non-clinical support staff including administration, cleaning, or laboratory personnel were excluded, unless clinical subgroups could be extracted independently. Studies must have used a validated assessment tool for PTSD, based off DSM-III-R to DSM-V, or ICD-10 to ICD-11. Cut-off scores with the highest sensitivities and specificities to indicate probable PTSD diagnosis were selected *a priori* based on evidence from published validation studies (see Supplementary Table 1), and any paper using a cut-off score outside of this range was excluded, regardless of the terminology used by authors to define scoring above cut-off. In cases where a study used multiple cut-off scores, the cut-off score with the highest diagnostic accuracy was used. In instances where studies had overlapping samples, only the most comprehensive study was included to avoid data duplication. This was determined based on sample size, or where participant demographics were described more thoroughly. In instances where data were reported across multiple timepoints within the same year, baseline data only were extracted. In instances where data were reported longitudinally across multiple years of COVID-19, data from the earliest collection point in each year were extracted. Longitudinal data over multiple years was only included in the by-year analysis and only baseline data from each study was included in the overall prevalence estimates for each time period (prior to COVID-19 or during COVID-19), to avoid duplication. The full exclusion criteria are listed in Supplementary Appendix 2.

### 2.3 Data extraction

Data extracted included study descriptions, participant demographics, recruitment methods, PTSD screening instrument, and prevalence data. Prevalence data were extracted as numerator (number of participants screening positive for PTSD) over denominator (total number of participants screened for PTSD). Where data were presented as numerator or denominator and percentage of participants screening positive, data were converted to numerator over denominator for analysis. No other assumptions were made about missing or unclear data and authors of primary studies were not contacted. The full list of extracted data is presented in Supplementary Appendix 2.

### 2.4 Assessment of data quality

The quality of each paper was assessed using the Joanna Briggs Institute (JBI) Critical Appraisal Checklist for Prevalence Studies (17). This consists of nine questions to which responses “yes,” “no” or “unclear” were given to each. A “yes” response was given a score of one, whilst a “no” or “unclear” response was given a score of zero. This resulted in a total quality assessment score from 0 to 9 for each paper. Studies with scores from 0 to 3 were considered low quality, from 4 to 6 were considered fair quality, and from 7 to 9 were considered high quality.

### 2.5 Data analysis

Data analysis was conducted using Comprehensive Meta-Analysis version 4 (CMA v4) software to calculate mean prevalence estimates with 95% confidence intervals (CIs). Studies were divided into subgroups by profession, and effect size was calculated within subgroups. Given the potential for heterogeneity, a random-effects model was used. Between-groups sub-analyses were conducted for year of data collection, profession (physicians, nurses, paramedics/EMTs, and other), economic status of country of study (high-income or low- to middle-income (18)), rate of COVID-19 deaths of country of study (>150 or <150 deaths per 100,000 population (5)), PTSD screening tool, quality of study (low, fair, or high quality) and studies with defined sampling frames and adequate response rates vs. those without. A meta-regression was run to assess the effect of time (year of data collection) prior to COVID-19 to account for secular trend bias in prevalence. Results were visualized using forest plots. Inter-rater reliability during screening was measured using Cohen’s *κ*. Heterogeneity was examined using the *I*^2^ statistic. Publication bias was assessed using the Egger test and visualized using funnel plots. Sensitivity analyses were conducted using the one-study removed approach. The significance level *α* was set at 0.05.

This systematic review is registered on the PROSPERO International Prospective Register of Systematic Reviews (CRD42022364955).

## 3 Results

**Figure 1:**
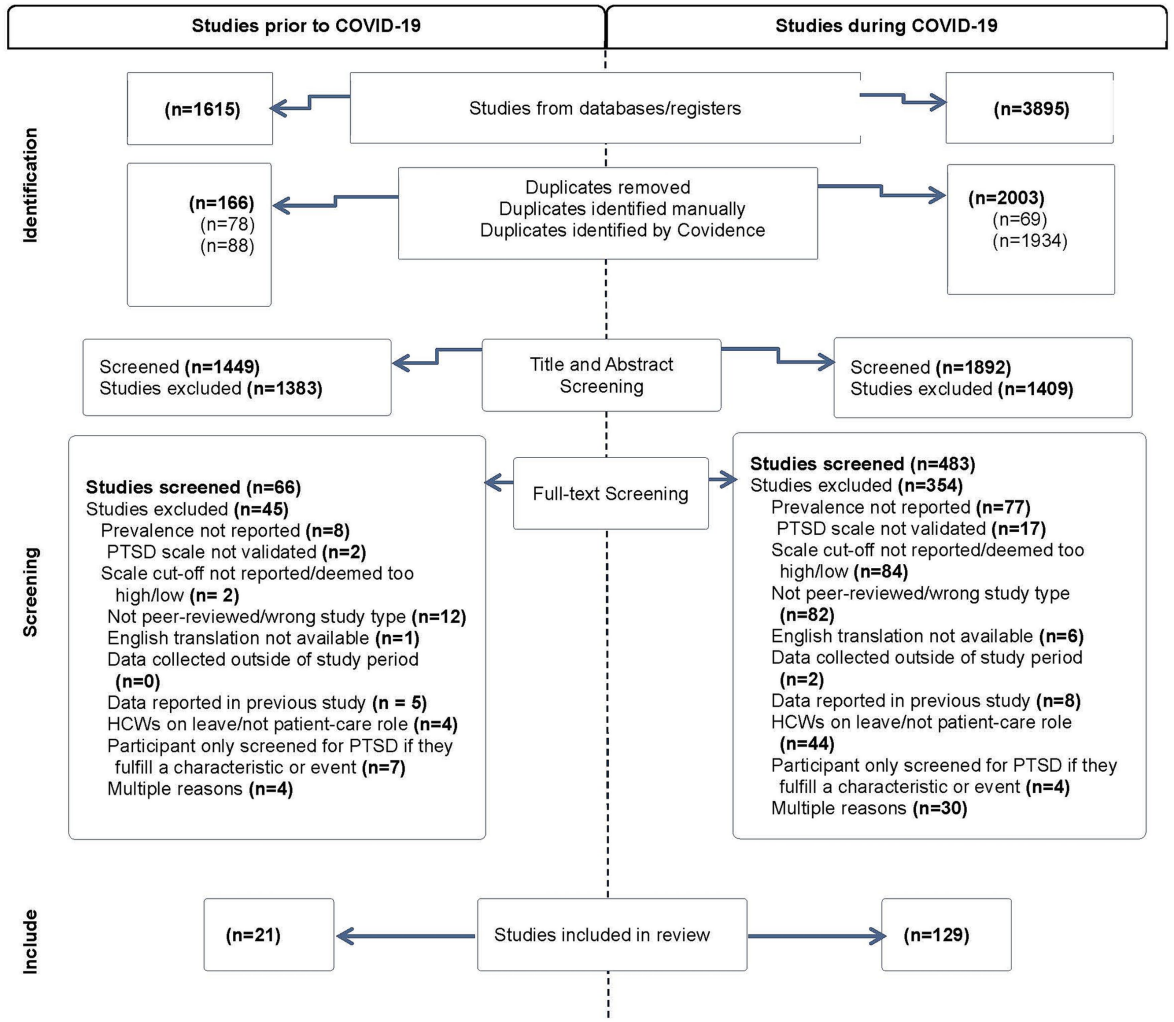
PRISMA flow diagram.

From the search of the three-year period leading up to the pandemic, 1,615 studies were initially retrieved. Of these, 166 studies were identified as duplicates, 1,383 were excluded in title and abstract screening, and 45 were excluded during full-text screening, resulting in 21 studies included in the meta-analysis. The search examining data during the COVID-19 pandemic retrieved 3,895 studies, of which 2,003 were removed as duplicates, 1,409 were excluded during title and abstract screening and 354 were excluded during full-text screening. This resulted in 129 eligible studies, as presented in Figure 1. Inter-rater reliability across both title and abstract screening and full-text screening was high, with Cohen’s *κ* ranging from 0.67 and 0.93 (see Supplementary Table 2).

Amongst the 21 studies conducted prior to the COVID-19 pandemic, data from 11,982 HCWs were reported. This included 257 nurses, 3,959 physicians, 3,805 paramedics/EMTs and 976 other patient-facing healthcare roles not otherwise stated, including allied health professionals, healthcare assistants, and student HCWs who spent at least 1 month on the wards during the pandemic. The remainder (*n* = 2,985) were reported as pooled data and not by profession.

The studies conducted during the pandemic comprised 130,363 HCWs, including 45,985 nurses, 39,524 physicians, 1,242 paramedics/EMTs and 7,246 other professions. The remainder (*n* = 36,366) were not stratified by profession. A total of 101 papers reported data from 2020 (109,493 total HCWs), 25 from 2021 (15,402 HCWs), and five from 2022 (5,468 HCWs). Two further papers specified that they were conducted during the COVID-19 pandemic but did not specify the dates of data collection. Five studies reported data longitudinally across 2 years. No studies reporting data from December 2019 or from 2023 were identified. All included studies are detailed in Table 1.

**Table 1:** Description of studies included in the meta-analysis.

Study ID	Country and setting	Country income level^a^	COVID-19 deaths per 100,000^b^	Study type	Profession groups extracted	Respondents (extracted professions only)	Year of data collection	% Female	PSTD instrument	Response rate
Prior to COVID-19										
Carleton 2018 (19)	Canada Public safety personnel in Canada (paramedics only extracted)	High	NA	Cross-sectional	Paramedic/EMT	5,813 (776)	2016	32.50%	PCL-5	5,813/NA
Cohen 2017 (20)	Israel Midwives across four medical centres in Israel	High	NA	Cross-sectional	Other	93	NA	NA	PSS-SR	93/203 (45.8%)
Colville 2017 (21)	United Kingdom Physicians and nurses across 2 ICUs in the UK	High	NA	Cross-sectional	Pooled (Nurse, Physician)	377	2012	83.40%	TSQ	334/744 (44.9%)
Eiche 2019 (22)	Germany Paramedics and emergency physicians across all German EMS stations	High	NA	Cross-sectional	Physician, Paramedic/EMT	2,684	2017	20.10%	Short Screening Scale for DSM-IV PTSD	2,684/NA
Hilton 2017 (23)	Canada Staff from one psychiatric hospital in Ontario	High	NA	Cross-sectional	Nurse	218 (~118)	NA	NA	PCL	218/~723 (30%)
Jackson 2019 (24)	USA Trauma and non-trauma surgeons via email from medical association databases	High	NA	Cross-sectional	Physician	1,026	2016	39.40%	PC-PTSD	1,026/6,957 (14.7%)
Jackson 2019 (25)	USA Resident physicians via email from medical association databases	High	NA	Cross-sectional	Physician	1,904	2016	60.00%	PC-PTSD	1,890/1,1860 (15.9%)
Kannan 2019 (26)	USA Resident physicians from three tertiary care institutions around Philadelphia	High	NA	Cross-sectional	Physician	194	NA	NA	PCL-5	194/280 (69.3%)
Kerai 2017 (27)	Pakistan Emergency medical staff from one emergency medical service in Karachi	Low-Middle	NA	Cross-sectional	Pooled (Physician, Nurse, Paramedic/EMT, Other)	507	2014	0%	IES-R	507/536 (94.6%)
Khazaei 2019 (28)	Iran EMTs in the Emergency Medical Services (EMS) of Hamadan province	Low-Middle	NA	Cross-sectional	Paramedic/EMT	259	2018	0%	PCL-5	259/307 (84.4%)
Kucmin 2018 (29)	Poland Paramedics employed by the Polish Emergency Medical Service in five voivodships	High	NA	Cross-sectional	Paramedic/EMT	159	NA	12.60%	IES-R	159/440 (36.1%)
Leinweber 2017 (30)	Australia Midwives recruited through professional body the Australian College of Midwives	High	NA	Cross-sectional	Other	601	2014	NA	PSS-SR	601/4,578 (13.1%)
Linane 2019 (31)	Ireland Junior physicians (interns and SHOs) in two teaching hospitals in Ireland	High	NA	Cross-sectional	Physician	110	2016	NA	PCL-6	110/298 (36.9%)
Luftman 2017 (32)	USA In hospital HCWs and pre-hospital providers recruited from four Trauma Regional Advisory Council (RAC) systems in Texas	High	NA	Cross-sectional	Nurse, Physician, Paramedic/EMT, Other	546 (492)	NA	NA	PC-PTSD	546/~2,500 (21.8%)
McFarland 2017 (33)	USA Internal medicine interns and residents working on rotation at a hospital in New York Longitudinal: pre-rotation and at end of rotation	High	NA	Longitudinal	Physician	56	2013	58.00%	IES-R	56/96 (58.3%)
Rodríguez-Rey 2019 (34)	Spain HCWs at 9 hospitals working in Paediatric ICU, and other paediatric units	High	NA	Cross-sectional	Pooled (Nurse, Physician, Other)	487	NA	83.20%	TSQ	487/NA
Rybojad 2019 (35)	Poland Emergency Medical Service employees across 20 medical services	High	NA	Cross-sectional	Pooled (Nurse, Physician, Paramedic/EMT, Other)	100	NA	28.0%	IES-R	100/200 (50%)
Schafer 2018 (36)	Germany Physicians and nurses working in anaesthesiology and ICU at one hospital	High	NA	Cross-sectional	Nurse, Physician	52	NA	65.40%	PCL-5	52/100 (52%)
Thompson 2017 (37)	UK Surgical trainees UK-wide distributed via emails from surgical trainee email distribution lists and social media	High	NA	Cross-sectional	Physician	144	2015	38.60%	IES-R	144/NA
van Steijn 2019 (38)	The Netherlands Paediatricians recruited via email from the Paediatric Association of The Netherlands	High	NA	Cross-sectional	Physician	381	2016	67.30%	TSQ	381/2,160 (17.6%)
Zhou 2018 (39)	China HCWs from 27 hospitals in the Heilongjiang province in China	Low-Middle	NA	Cross-sectional	Pooled (Nurse, Physician, Other)	1,557	2015	75.90%	PCL-C	1,557/1,679 (92.7%)
During COVID-19										
Amsalem 2021 (40)	USA HCWs from online survey	High	>150	Longitudinal	Pooled (Nurse, Physician, Paramedic/EMT, Other)	350	2020	74.0%	PC-PTSD-5	350/NA
Arnetz 2020 (41)	USA 3 nationwide nursing associations	High	>150	Cross-Sectional	Nurse	632	2020	93.6%	PCL-6	632/NA
Bani Issa 2022 (42)	United Arab Emirates Nurses from 3 government hospitals in northern UAE	High	<150	Cross-Sectional	Nurse	370	2020	84.1%	PDS-5	370/450 (82.2%)
Bassi 2021 (43)	Italy Nurse and physician associations in Lombardy	High	>150	Cross-Sectional	Pooled (Nurse, Physician, Other)	653	2020	73.8%	PCL-5	653/NA
Bates 2021 (44)	UK ICU staff from 1 UK teaching hospital	High	>150	Cross-Sectional	Nurse, Physician, Other	93	2020	77.0%	PCL-5	93/196 (47.4%)
Baumann 2021 (45)	USA Emergency physicians from 7 residencies and affiliated institutions	High	>150	Longitudinal	Physician	259	2020	49.8%	PC-PTSD-5	259/426 (61.5%)
Blanco-Daza 2022 (46)	Spain Nursing staff from one hospital in Madrid	High	>150	Cross-Sectional	Nurse	344 (342)	2020	88.7%	DTS	344/3,149 (10.9%)
Bonzini 2022 (47)	Italy All workers from one large general hospital in Milan	High	>150	Cross-Sectional	Nurse, Physician, Other	990 (712)	2021	70.0%	IES-R	990/1,610 (61.5%)
Caillet 2020 (48)	France ICU caregivers from one teaching hospital in Lyon	High	>150	Cross-Sectional	Nurse, Physician, Other	195 (182)	2020	75.0%	IES-R	195/219 (89.0%)
Carola 2022 (49)	Italy HCWs in the COVID-19 ICU at one university hospital in Rome	High	>150	Cross-Sectional	Pooled (Nurse, Physician)	35	2021	24.3%	IES-R	35/NA
Chang 2022 (50)	USA Postgraduate years 1–4 emergency medicine residents from one academic healthcare system	High	>150	Longitudinal	Physician	31	2020	NA	PCL-5	31/60 (51.7%)
Chatzittofis 2021 (51)	Cyprus HCWs recruited through the Cyprus Medical Association, the Professional Association of Physiotherapists and social networks	High	<150	Cross-Sectional	Nurse, Physician, Other	424	2020	58.5%	IES-R	424/NA
Chauhan 2021 (52)	India HCWs from two large tertiary care hospitals	Low-middle	<150	Cross-Sectional	Pooled (Nurse, Physician, Paramedic/EMT)	400	2020	36.3%	IES-R	400/NA
Che 2022 (53)	China Anaesthesiologists nationwide recruited through Chinese Society of Anaesthesiology	Low-middle	<150	Cross-Sectional	Physician	6,631	2021	59.9%	IES-R	6,631/8,850 (74.9%)
Chen 2020 (54)	China Staff from one university hospital in Xuzhou city	Low-middle	<150	Cross-Sectional	Nurse, Physician, Other	171 (152)	NA	67.8%	PCL-C	171/198 (86.3%)
Cobo-Cuenca 2022 (55)	Spain Nursing graduates in first year of work from one Spanish university	High	>150	Longitudinal	Nurse	92	2020	89.1%	DTS-8	92/400 (23.0%)
Conti 2020 (56)	Italy HCWs nationwide recruited through social networks	High	>150	Cross-Sectional	Nurse, Physician, Other	877 (831)	2020	76.5%	IES-R	877/NA
Costantini 2022 (57)	Italy Hospital workers from one hospital in Rome	High	>150	Cross-Sectional	Nurse, Physician, Other	324 (244)	2020	78.1%	IES-R	324/NA
Couper 2022 (58)	UK Nurses and Midwives recruited through nursing and midwifery organisations and media	High	>150	Longitudinal	Pooled (Nurse, Other)	2,040	2020	91.6%	IES-R	2,040/NA
Crowe 2021 (59)	Canada Nurses working in ICU or HAU at one large teaching hospital	High	<150	Cross-Sectional	Nurse	108	2020	89.9%	IES-R	108/~242 (44.6%)
D’Alessandro-Lowe 2023 (60)	Canada Respiratory therapists recruited through Canadian Society for Respiratory Therapists	High	<150	Cross-Sectional	Other	218	2021	84.9%	PCL-5	218/4,875 (4.5%)
Damico 2022 (61)	Italy ICU nurses across 10 nationwide hospitals longitudinally across 2020 and 2021	High	>150	Longitudinal	Nurse	2020: 359 2021: 359	2020/2021	35.1%	PCL-C	2020: 359/480 (74.8%) 2021: 359/480 (74.8%)
Dehon 2021 (62)	USA Emergency physicians across 11 institutions nationwide	High	>150	Cross-Sectional	Physician	255	2020	37.1%	PCL-5	255/517 (49.3%)
de Lima Osório 2023 (63)	Brazil HCWs recruited through councils, health institutions and media	Low-middle	>150	Longitudinal	Pooled (Nurse, Physician, Other)	Baseline 2020: 916 2021 follow-up: 177	2020	79.7%	PCL-5	916/NA, 177/NA
Demartini 2020 (64)	Italy Online survey of the general public and HCWs	High	>150	Cross-Sectional	NA	432 (123)	2020	79.5%	IES-R	432/NA
Di Tella 2020 (65)	Italy Snowball sampling of HCWs in Piedmont, Italy	High	>150	Cross-Sectional	Pooled (Nurse, Physician)	145	2020	72.4%	PCL-5	145/NA
Dykes 2022 (66)	UK Single-centre ICU HCWs	High	>150	Cross-Sectional	Nurse, Physician, Other	131 (126)	2020	74.0%	IES-R	131/NA
Engelbrecht 2021 (67)	South Africa All nurses working in the Free State province	Low-middle	>150	Cross-Sectional	Nurse	286	2021	86.7%	IES-R	286/5,209 (5.5%)
Fattori 2021 (68)	Italy Hospital workers from one university hospital in Milan	High	>150	Longitudinal	Nurse, Physician, Other	550 (419)	2020	64.2%	IES-R	550/NA
Feingold 2021 (69)	USA HCWs in one tertiary care hospital in New York City	High	>150	Cross-Sectional	Nurse, Physician, Other	2,574 (2,410)	2020	73.6%	PCL4-5	2,574/6,026 (42.7%)
Fournier 2022 (70)	France Medical and non-medical professionals across 77 hospitals	High	>150	Cross-Sectional	Nurse, Physician, Other	4,187 (2,803)	2020	81.7%	IES-R	4,187/NA
Gainer 2021 (71)	USA Physicians recruited through email and social media nationwide	High	>150	Cross-Sectional	Physician	~1,717	2020	56.1%	PCL-6	1,717/NA
Geng 2021 (72)	China Medical staff across 4 designated hospitals	Low-middle	<150	Cross-Sectional	Pooled (Nurse, Physician, Other)	317	2020	69.7%	PCL-5	317/481 (65.9%)
Ghio 2021 (73)	Italy Personnel across multiple hospitals in Galliera	High	>150	Longitudinal	Nurse, Physician, Other	731	2020	76.1%	IES-R	731/2,461 (29.7%)
González-Mesa 2021 (74)	Spain Obs-Gyns specialists recruited from Spanish Society of Obstetrics and Gynaecology (SEGO)	High	>150	Cross-Sectional	Physician	220	2021	68.1%	ITQ	220/800 (27.5%)
Gonzalez Mendez 2022 (75)	China Health personnel at multiple facilities across 7 regions of China	Low-middle	<150	Cross-Sectional	Nurse, Physician	1,263 (969)	2021	77.4%	PC-PTSD-5	1,263/NA
Gorini 2021 (76)	Italy HCWs in several hospitals and healthcare institutions	High	>150	Cross-Sectional	Nurse, Physician, Other	671 (624)	2021	71.7%	IES-R	671/NA
Greenberg 2021 (77)	UK ICU HCWs from 9 UK hospitals	High	>150	Cross-Sectional	Nurse, Physician, Other	709	2020	NA	PCL-6	709/NA
Guardiano 2022 (78)	USA Community nurses through nursing organisations and prison nurses from one Californian prison	High	>150	Cross-Sectional	Nurse	93	2020	91.0%	PCL-6	93/NA
Guo 2021 (79)	China Workers across 8 hospitals in Wuhan and other cities	Low-middle	<150	Cross-Sectional	Nurse, Physician, Other	1,091 (756)	2020	67.4%	PCL-C	1,091/1,805 (60.4%)
Guttormson 2022 (80)	USA Critical care nurses recruited through social media and American Association of Critical Care Workers	High	>150	Cross-Sectional	Nurse	360	2020	88.1%	TSQ	360/NA
Habtamu 2021 (81)	Ethiopia Frontline HCWs at a COVID-19 treatment centre in Addis Ababa	Low-middle	<150	Cross-Sectional	Pooled (Nurse, Physician, Other)	238	2020	56.3%	PCL-C	238/280 (85.5%)
Havaei 2021 (82)	Canada Nurses through Provincial Nurses Union email and social media	High	<150	Cross-Sectional	Nurse	3,676	2020	NA	PTSS-14	3,676/48,000 (7.7%)
Heesakkers 2021 (83)	The Netherlands Nationwide through Dutch Association of ICU nurses and a non-profit organisation for critical care medicine	High	<150	Cross-Sectional	Nurse	726	2020	73.8%	IES-6	726/NA
Heesakkers 2023 (84)	The Netherlands Nationwide through Dutch Association of ICU nurses and a non-profit organisation for critical care medicine	High	<150	Cross-Sectional; Longitudinal	Nurse	589	2021	73.8%	IES-6	589/NA
Hendrickson 2022 (85)	USA Frontline HCWs and first responders recruited online	High	>150	Cross-Sectional	Nurse, Physician, Paramedic/EMT, Other	402 (288)	2020	NA	PCL-5	402/NA
Hernandez 2021 (86)	USA Nurses recruited from social media	High	>150	Cross-Sectional	Nurse	298	2020	96.7%	TSQ	298/NA
Hickling 2022 (87)	USA Nurses recruited via email and social media	High	>150	Cross-Sectional	Nurse	112	2020	92.0%	PCL-5	112/NA
Hill 2021 (88)	USA Nurses from two acute care hospitals in Louisiana	High	>150	Cross-Sectional	Nurse	31	2020	NA	PDS-5	31/50 (62.0%)
Honarmand 2022 (89)	Canada Hospital workers (Physicians and nurses extracted only) from 12 hospitals in Ontario	High	<150	Cross-Sectional	Nurse, Physician	849 (434)	2020	87.7%	IES-R	849/NA
Ibrahim 2023 (90)	Belgium Physiotherapists recruited from 12 hospitals in Brussels	High	>150	Cross-Sectional	Other	115	2022	55.7%	PCL-5	115/828 (13.9%)
Iqbal 2021 (91)	Pakistan Medics and paramedics–no recruitment information given	Low-middle	<150	Cross-Sectional	Physician, Paramedic/EMT	360	2020	48.9%	PCL-C	360/NA
James 2022 (92)	USA HCWs recruited from the Eastern Association for the Surgery of Trauma (EAST)	High	>150	Cross-Sectional	Nurse, Physician, Other	393 (392)	2020	39.4%	PCL-6	393/2,302 (17.1%)
Johns 2022 (93)	UK Physicians and final year medical students recruited via email and social media	High	>150	Cross-Sectional	Pooled (Physician, Other)	346	2020	75.0%	PCL-5	346/NA
Jovarauskaite 2022 (94)	Lithuania Nurses recruited via healthcare institutions and social media	High	>150	Cross-Sectional	Nurse	206	2021	97.1%	ITQ	206/NA
Kabunga 2021 (95)	Uganda Nurses recruited from Ugandan Nurses and Midwives Council (UNMC) from 9 hospitals in central Uganda	Low-middle	<150	Cross-Sectional	Nurse	601	2021	60.6%	PCL-C	601/~637 (94.4%)
Kachadourian 2021 (96)	USA Frontline HCWs from one hospital in New York City	High	>150	Cross-Sectional	Nurse, Physician, Paramedic/EMT, Other	~2,571	2020	73.6%	PCL4-5	2,571/6,026 (43.7%)
Kader 2021 (97)	Qatar ICU physicians and nurses from one hospital in Qatar	High	<150	Cross-Sectional	Nurse, Physician	124	2020	42.8%	PDS-5	124/143 (86.7%)
Kalyanaraman 2022 (98)	USA Paediatric critical care physicians recruited from the Society of Critical Care Medicine and the American Academy of Paediatricians	High	>150	Cross-Sectional	Physician	294	2020	53.7%	PCL-5	294/1,843 (15.9%)
Kim 2022 (99)	South Korea Paramedics and EMTs from public emergency medical services and fire departments in each Korean Province	High	<150	Cross-Sectional	Paramedic/EMT	326	2020	32.8%	PC-PTSD-5	326/NA
King 2022 (100)	Ireland Mental health nurses from 3 in-patient centres in Dublin	High	>150	Cross-Sectional	Nurse	119	2020	82.0%	IES-R	119/300 (39.7%)
Korkut 2022 (101)	Turkey Staff from one pandemic hospital	Low-middle	<150	Cross-Sectional	Nurse, Physician	300 (100)	2020	56.7%	SCID-5	300/NA
Kumar 2023 (102)	India Paediatric ICU/COVID-19 HCWs at one tertiary care hospital in north India	Low-middle	<150	Cross-Sectional	Nurse, Physician, Other	184 (180)	2021	51.1%	TSQ	184/230
Kwobah 2021 (103)	Kenya HCWs recruited through social media	Low-middle	<150	Cross-Sectional	Pooled (Nurse, Physician, Other)	348	2020	54.5%	PC-PTSD-5	348/NA
Lamb 2021 (104)	UK Staff working across 3 London-based health service trusts	High	>150	Cross-Sectional	Nurse, Physician, Other	2,447 (1,484)	2020	74.8%	PCL-6	2,447/3,7,870 (6.5%)
Laurent 2022 (105)	France Frontline HCWs working in ICU at 70 hospitals nationwide across France	High	>150	Cross-Sectional	Nurse, Physician, Other	1,758	2020	75.0%	IES-R	1,758/NA
Lee 2021 (106)	South Korea All hospital employees in South Chungcheong province	High	>150	Cross-Sectional	Pooled (Nurse, Physician, Other)	396 (306)	NA	80.3%	PCL-5	396/631 (62.8%)
Lee 2022 (107)	Taiwan Nurses and physicians at one hospital	High	<150	Cross-Sectional	Pooled (Nurse, Physician)	354	2021	82.8%	IES-R	354/NA
Lei 2021 (108)	China General public surveyed through chat service and HCWs extracted	Low-middle	<150	Cross-Sectional	NA	1,593 (251)	2020	61.3*	PCL	1,593/NA
Levi 2022 (109)	USA ICU nurses at an academic health science centre in the southeastern United States	High	>150	Cross-Sectional	Nurse	10	2020	90.0%	PCL-5	10/NA
Li 2021 (110)	China Frontline nurses at one hospital in Anhui	Low-middle	<150	Longitudinal	Nurse	356	2020	86.2%	PCL-5	356/362 (98.3%)
Li 2022A (111)	USA Nursing alumni from a large midwestern university	High	>150	Cross-Sectional	Nurse	124	2020	90.6%	PCL-5	124/6,000 (2.1%)
Li 2022B (112)	China Frontline HCWs in China	Low-middle	<150	Cross-Sectional	Nurse, Physician, Other	938	2022	93.7%	PCL-C	938/NA
Liu 2023 (113)	China Frontline HCWs involved in COVID-19 management in Shaanxi region	Low-middle	<150	Cross-Sectional	Nurse, Physician, Other	3,455	2022	83.1%	PCL-5	3,455/NA
Lixia 2022 (114)	China HCWs from 46 hospitals designated by the Chongqing Municipal Health Commission	Low-middle	<150	Cross-Sectional	Nurse, Physician, Other	33,706 (31,200)	2020	76.7%	PCL-C	33,706/51,637 (65.3%)
Lombard 2022 (115)	South Africa Anaesthetists recruited via email through South African Society of Anaesthesiologists (SASA)	Low-middle	>150	Cross-Sectional	Physician	391	2020	48.8%	PCL-5	391/2,028 (19.3%)
Lopez-Salinas 2023 (116)	Mexico Frontline Healthcare Workers in a COVID-19 Hospital in Monterrey, Northeast Mexico	Low-middle	>150	Cross-Sectional	Nurse, Physician	131	2020	63.4%	IES-R	131/325 (40.3%)
Lu 2021 (117)	Taiwan Frontline HCWs at one university hospital in Tainan, Taiwan	High	<150	Cross-Sectional	Pooled (Nurse, Physician, Other)	500	2020	91.6%	IES-6	500/~5,000 (10.0%)
Marcomini 2021 (118)	Italy All registered nurses at one hospital in Lombardy	High	>150	Cross-Sectional	Nurse	173	2020	76.3%	IES-R	173/275 (62.9%)
Marsden 2022 (119)	Australia Nurses and midwives in Tasmania longitudinally (2020–2021) from the Tasmanian Health Service email list	High	<150	Longitudinal	Nurse, Other	Survey 1 (2020):684 / Survey 3 (2021): 439	2020/2021	88.1% / 90.1%	IES-R	Baseline 2020: 684/4,884 (14.0%) Follow-up 2021: 439/4,884 (9.0%)
Martínez-Caballero 2021 (120)	Spain Emergency medical workers recruited via email to the emergency institutions of Castile and Leon and Madrid	High	>150	Cross-Sectional	Nurse, Physician, Paramedic/EMT	317	2020	46.4%	DTS-8	317/~843 (37.6%)
Martsenkovskyi 2022 (121)	Ukraine Physicians and nurses recruited through social media and snowball sampling	Low-middle	>150	Cross-Sectional	Nurse, Physician	330	2021	80.3%	PCL-5	330/NA
McGuinness 2022 (122)	Australia HCWs recruited through healthcare organisations, primarily in Melbourne, Victoria	High	<150	Longitudinal; baseline study of a longitudinal project	Nurse, Physician, Paramedic/EMT, Other	984 (742)	2021	72.8%	IES-6	984/~50,000 (2.0%)
McGuinness 2023 (123)	Australia Longitudinal study (2021–2022) of HCWs through healthcare organisations, primarily in Melbourne, Victoria	High	<150	Longitudinal	Nurse, Physician, Paramedic/EMT, Other	993 (837)	2021–2022 Data extracted from 2022 only	72.6%	IES-6	993/~50,000 (2.0%)
Mehta 2022 (124)	Canada ICU HCWs, invitations to all ICU directors across Canada and professional organisations	High	<150	Cross-Sectional	Nurse, Physician, Other	375 (291)	2020	80.2%	IES-R	375/NA
Minelli 2022 (125)	Italy Mental healthcare workers in Italy recruited through snowball sampling	High	>150	Cross-Sectional	Nurse, Physician, Other	271	2020	73.1%	IES-R	217/NA
Mohsin 2022 (126)	Pakistan Physicians from one tertiary care hospital in Rawalpindi	Low-middle	<150	Cross-Sectional	Physician	173	2022	48.0%	IES-R	173/NA
Mosheva 2021 (127)	Israel Physicians and nurses at a large tertiary medical centre in central Israel	High	<150	Cross-Sectional	Physician, Nurse	823	2020	67.2%	PC-PTSD-5	823/3,625 (22.7%)
Murata 2021 (128)	USA General population (Adults, Adolescents and HCWs) recruited through Facebook, University of Pittsburgh, and other health care systems around Pittsburgh	High	>150	Cross-Sectional	NA	2,338 (940)	2020	80.0%	PC-PTSD-5	2,338/NA
Ng 2022A (129)	Australia General practitioners through media, social media, and emailing healthcare facilities	High	<150	Cross-Sectional	Physician	389	2020	81.0%	IES-6	389/NA
Ng 2022B (130)	Australia Frontline perioperative healthcare staff in a tertiary public hospital–longitudinal in 2020	High	<150	Longitudinal	Pooled (Nurse, Physician, Other)	193 longitudinally (95 at baseline)	2020	60.1%	PC-PTSD-5	95/~300 (31.7%)
Ouazzani Housni Touhami 2022 (131)	Morocco Medical physicians recruited through personal Facebook, WhatsApp, and email	Low-middle	<150	Cross-Sectional	Physician	1,267	2020	59.2%	PCL-5	1,267/~2,000 (63.4%)
Ouyang 2022 (132)	China Longitudinal (2020–2021) study of HCWs recruited from designated hospitals	Low-middle	<150	Longitudinal	Pooled (Nurse, Physician, Other)	Baseline 2020: 317 / Follow-up 2021: 403	2020/2021	69.7% / 66.7%	PCL-5	Baseline: 317/481 (65.9%) Follow-up 403/523 (77.1%)
Öz Tunçer 2022 (133)	Turkey Paediatrics neurologists nationwide	Low-middle	<150	Cross-Sectional	Physician	222	2020	73.3%	IES-R	232/405 (54.8%)
Pan 2021 (134)	China HCWs recruited through chat service in Wuhan	Low-middle	<150	Cross-Sectional	Nurse, Physician	659 (628)	2020	90.6%	PCL-5	659/NA
Pappa 2021 (135)	Greece HCWs across 6 COVID-19 hospitals	High	>150	Cross-Sectional	Pooled (Nurse, Physician, Other)	434	2020	68.8%	IES-R	434/NA
Pascoe 2022A (136)	Australia Nurses recruited through healthcare facilities and media, primarily in Victoria	High	<150	Cross-Sectional	Nurse	~3,056	2020	88.9%	IES-6	~3,056/NA
Pascoe 2022B (137)	Australia Senior and junior physicians recruited through hospitals and media, primarily in Victoria	High	<150	Cross-Sectional	Physician	1,958	2020	63.6%	IES-6	1,958/NA
Pasin 2020 (138)	Italy Frontline residents in Anaesthesia, Intensive Care and Emergency Medicine across Italy, recruited from social media/email	High	>150	Cross-Sectional	Physician	312	2020	56.8%	IES-R	312/1,297 (24.1%)
Qureshi 2020 (139)	Pakistan HCWs in tertiary care hospitals recruited through online messaging service	Low-middle	<150	Cross-Sectional	Pooled (Nurse, Physician, Other)	171	2020	62.6%	IES-R	171/NA
Qutishat 2021 (140)	Jordan Nurses working with COVID-19 patients recruited through social media	Low-middle	<150	Cross-Sectional	Nurse	259	2020	47.9%	PCL-5	259/NA
Reid 2022 (141)	Norway Ambulance personnel recruited through the Regional Health Trust of Central Norway	High	<150	Cross-Sectional	Nurse, Paramedic/EMT, Other	479	2021	47.2%	PTSS-10	479/1,052 (45.5%)
Renzi 2023 (142)	Italy Nurses recruited through social media	High	>150	Cross-Sectional	Nurse	400	2021	78.7%	IES-R	400/NA
Roberts 2021 (143)	United Kingdom and Ireland Physicians surveyed over 3 timepoints, recruited via email or messaging groups through medical networks	High	>150	Longitudinal	Physician	2,730	2020	51.0%	IES-R	2,730/5,440 (50.2%)
Sar-El 2022 (144)	Israel First year medical interns working at one facility in Tel-Aviv	High	<150	Cross-Sectional	Physician	115	2021	49.3%	PCL-5	115/188 (61.2%)
Seifeldin Abdeen 2023 (145)	Egypt Physicians recruited via social media	Low-middle	<150	Cross-Sectional	Physician	124	2020	64.5%	PCL-C	124/NA
Shechter 2022 (146)	USA HCWs from one medical facility in New York City, surveyed at 2-week intervals for 10 weeks	High	>150	Longitudinal	Pooled (Nurse, Physician, Other)	Baseline 230	2020	79.6%	PC-PTSD	230/NA
Sobregrau Sangra 2022 (147)	Spain Frontline HCWs from two Spanish tertiary hospitals	High	>150	Cross-Sectional	Pooled (Nurse, Physician, Other)	184	2020	84.8%	PCL-5	184/NA
Somi 2022 (148)	Iran Health professional from one university in Tabriz–HCWs involved in treatment only extracted	Low-middle	>150	Cross-Sectional	Pooled (Nurse, Physician, Other)	298 (61)	2021	64.4%	PC-PTSD-5	298/NA
Song 2020 (149)	China Physicians and nurses nationwide working in hospitals involved in the treatment of COVID-19	Low-middle	<150	Cross-Sectional	Pooled (Nurse, Physician)	14,825	2020	64.3%	PCL-5	14,825/NA
Stafseth 2022 (150)	Norway Nurses and physicians caring for COVID-19 patients across 28 hospitals with a COVID-ICU	High	<150	Cross-Sectional	Nurse, Physician	484	2020	77.9%	PCL-5	484/NA
Stanislawski 2023 (151)	USA Third-year Medical Students rotating through wards in New York City, 4 longitudinal timepoints (2020–2021)	High	>150	Longitudinal	Other	Survey 1 after clinical placements (2020): 82 First survey of 2021: 77	2020/2021	45.7%	PCL-5	82/147 (55.8%) 77/147 (52.4%)
Styra 2021 (152)	Canada HCWs recruited via email from 2 tertiary and 2 community care hospitals in Toronto	High	<150	Cross-Sectional	Nurse, Physician, Other	3,357 (2,430)	2020	84.2%	IES-R	3,357/NA
Tatsuno 2021 (153)	Japan Nurses in acute care units, recruited from medical organisations, mailing lists and social media	High	<150	Cross-Sectional	Nurse	333	2020	64.4%	IES-6	333/NA
Villalba-Arias 2021 (154)	Paraguay HCWs from 5 selected hospitals via instant messaging service	Low-middle	>150	Cross-Sectional	Nurse, Physician, Other	432 (389)	2020	71.1%	PCL-C	432/886 (48.8%)
Wang 2020 (155)	China Nurses from 3 tertiary hospitals in Hubei provinces	Low-middle	<150	Cross-Sectional	Nurse	202	2020	87.6%	PCL-C	202/211 (95.7%)
Wanigasooriya 2021 (156)	UK All hospital staff from ten NHS acute general and mental health hospitals	High	>150	Cross-Sectional	Nurse, Physician	2,638 (1,235)	2020	79.5%	IES-R	2,638/NA
Wild 2022 (157)	UK Frontline HCWs from 4 hospital and ambulance trusts	High	>150	Cross-Sectional	Pooled (Nurse, Physician, Paramedic/EMT, Other)	103	2020	55.0%	PCL-5 then SCID-5 for those screening positive	103/NA
Wojcik 2022 (158)	USA Compared sexual and gender minority (SGM) frontline HCWs to non-SGM frontline HCWs at a hospital in New York	High	>150	Cross-Sectional	NA	674	2020	67.1%	PC-PTSD-5	674/NA
Yalçın 2020 (159)	Turkey HCWs (physicians and nurses) and non-HCWs working at one paediatric hospital	Low-middle	<150	Cross-Sectional	Nurse, Physician	257 (147)	2020	43.6%	IES-R	257/1,100 (23.4%)
Yang 2022 (160)	China HCWs from hospitals in seven cities of Heilongjiang Province in China	Low-middle	<150	Cross-Sectional	Nurse, Physician, Other	1,993 (1,593)	2020	83.1%	PCL-5	1,993/2,260 (88.2%)
Yeo 2021 (161)	South Korea Physicians and Nurses from 43 emergency departments in Daegu and Gyeongbuk	High	<150	Cross-Sectional	Nurse, Physician	520	2020	63.8%	PC-PTSD-5	520/1,116 (46.6%)
Yin 2020 (162)	China HCWs recruited through snowball sampling online and through email and chat services	Low-middle	<150	Cross-Sectional	Nurse, Physician	371 (331)	2020	61.5%	PCL-5	371/NA
Yin 2021 (163)	China Medical staff at a general hospital in Shanghai	Low-middle	<150	Cross-Sectional	Pooled (Nurse, Physician, Other)	304	2020	93.4%	PCL-5	304/400 (76.0%)
Zakeri 2021 (164)	Iran Frontline nurses at one hospital	Low-middle	>150	Cross-Sectional	Nurse	185	2020	77.3%	IES-R	185/300 (61.7%)
Zhang 2020 (165)	China Physicians and nurses at one hospital in Wuhan	Low-middle	<150	Cross-Sectional	Nurse, Physician	678 (642)	2020	85.1%	PCL-C	678/4,300 (15.8%)
Zhang 2021 (166)	China Medical HCWs and auxiliary staff at hospitals recruited using snowball sampling (medical HCWs extracted only)	Low-middle	<150	Cross-Sectional	Pooled (Nurse, Physician, Other)	401 (351)	2020	69.1%	IES-R	401/NA
Zhang 2022A (167)	China Nurses from 14 hospitals in Zhejiang Province, with 100 nurses aiding Wuhan	Low-middle	<150	Cross-Sectional	Nurse	200	2021	88.0%	IES-R	200/NA
Zhang 2022B (168)	China Physicians and nurses from paediatric ICUs in 62 hospitals across 31 provinces	Low-middle	<150	Cross-Sectional	Pooled (Nurse, Physician)	2,109	2020	85.0%	IES-R	2,109/3,055 (69.0%)

a High-income or low- to middle-income in country of study, as defined by World Bank Group (18).

b COVID-19 mortality rates per 100,000 population in country of study, according to the World Health Organisation (5).

DSM-5, Diagnostic and Statistical Manual of Mental Disorders 5; DTS, Davidson Trauma Scale; DTS-8, 8-item DTS; EMT, Emergency Medical Technician; IES-R, Impact of Event Scale – Revised; IES-6, 6-item Impact of Event Scale; ITQ, International Trauma Questionnaire; NA, Not Applicable; PCL, PTSD Checklist; PCL4-5, 4-item PCL-5; PCL-5, PCL for DSM-5; PCL-6, 6-item PCL; PCL-C, PCL-Civilian; PC-PTSD, Primary Care-PTSD Screen; PC-PTSD-5, PC-PTSD for DSM-5; PDS-5, Post-traumatic Diagnostic Scale for DSM-5; PSS-SR, PTSD Symptom Scale – Self-Report Version; PTSD, Post-traumatic stress disorder; PTSS-10, 10-item Post-traumatic Stress Scale; PTSS-14, 14-item Post-traumatic Stress Scale; SCID-5, Structural Clinical Interview for DSM-5 Disorders; TSQ, Trauma Screening Questionnaire.

**Figure 2:**
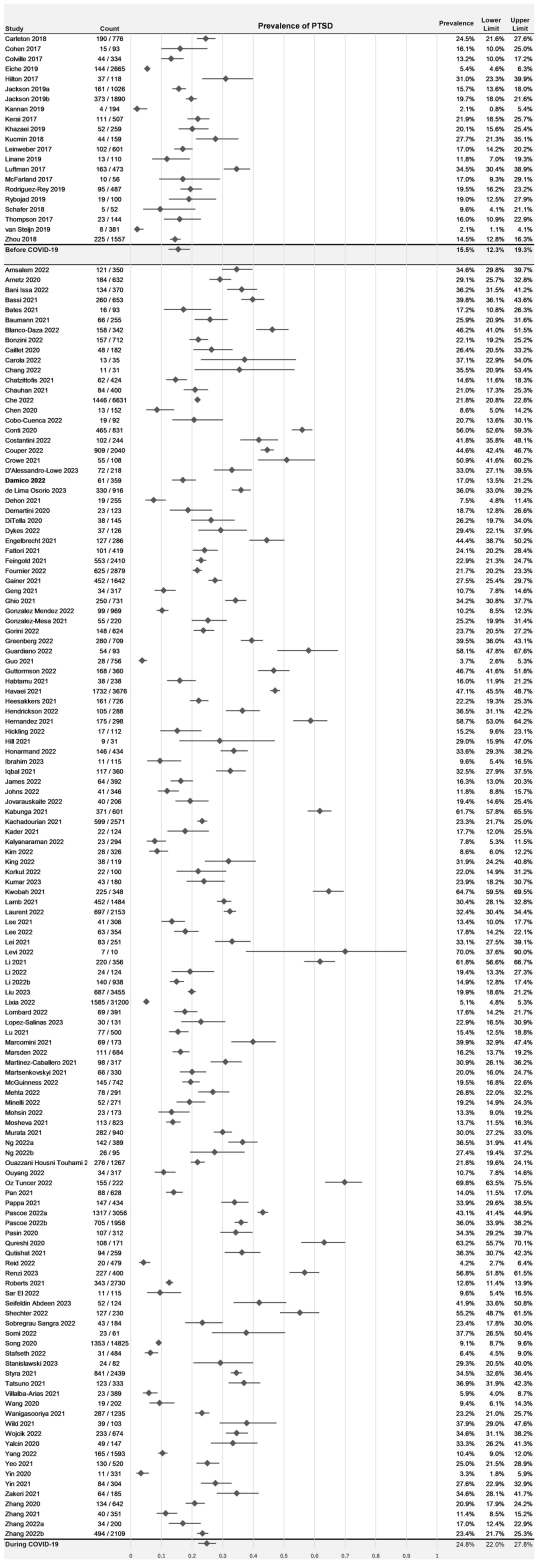
Prevalence estimates for individual studies included in the meta-analysis, stratified by studies prior to COVID-19 and studies during COVID-19. PTSD denotes post-traumatic stress disorder.

**Figure 3:**
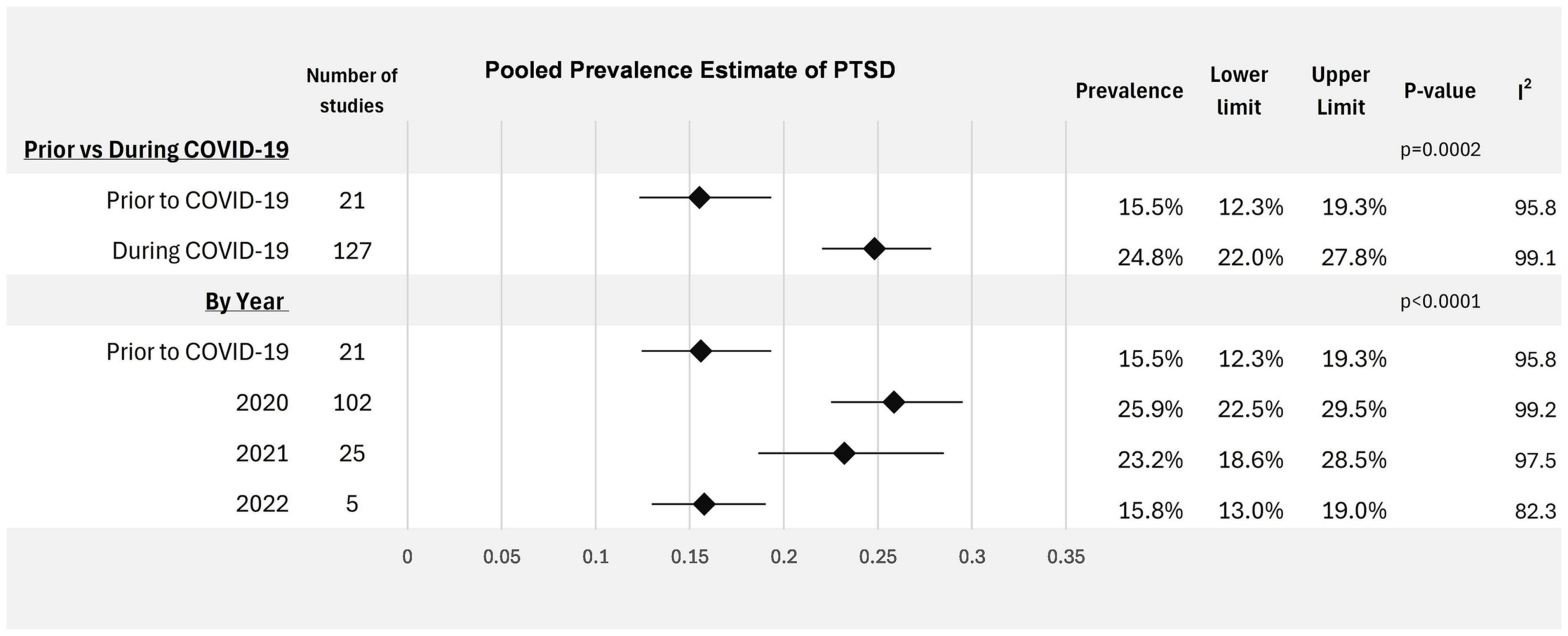
Forest Plot visualizing prevalence of probable PTSD in healthcare workers across periods of time. PTSD denotes post-traumatic stress disorder.

The overall estimated mean prevalence of probable PTSD in HCWs prior to the pandemic was 15.5% (95% CI: 12.3–19.3%, *I*^2^ = 95.8%). This increased significantly during the COVID-19 pandemic to 24.8% (95% CI: 22.0–27.8%, *I*^2^ = 99.1%; *p* = 0.0002), demonstrated in Figure 2. Prevalence estimates differed significantly across the time periods investigated (*p* < 0.0001), demonstrated in Figure 3. In 2020, the first year of the pandemic, the pooled prevalence estimate was highest at 25.9% (95% CI: 22.5–29.5%), which fell to 23.2% in 2021 (95% CI: 18.7–28.5%), and dropped again to 15.8% in 2022 (95% CI: 13.0–19.0%).

In order to investigate potential risk factors for higher rates of probable PTSD during the pandemic, several subgroups were examined. By profession, nurses showed the highest prevalence of PTSD at 26.9% (95% CI: 22.6–31.6%), followed by other healthcare workers at 21.2% (95% CI: 16.2–27.3%), then paramedics/EMTs at 20.5% (95% CI: 10.4–36.5%). Physicians showed the lowest prevalence during the pandemic at 17.2% (95% CI: 14.2–20.6%).

**Figure 4:**
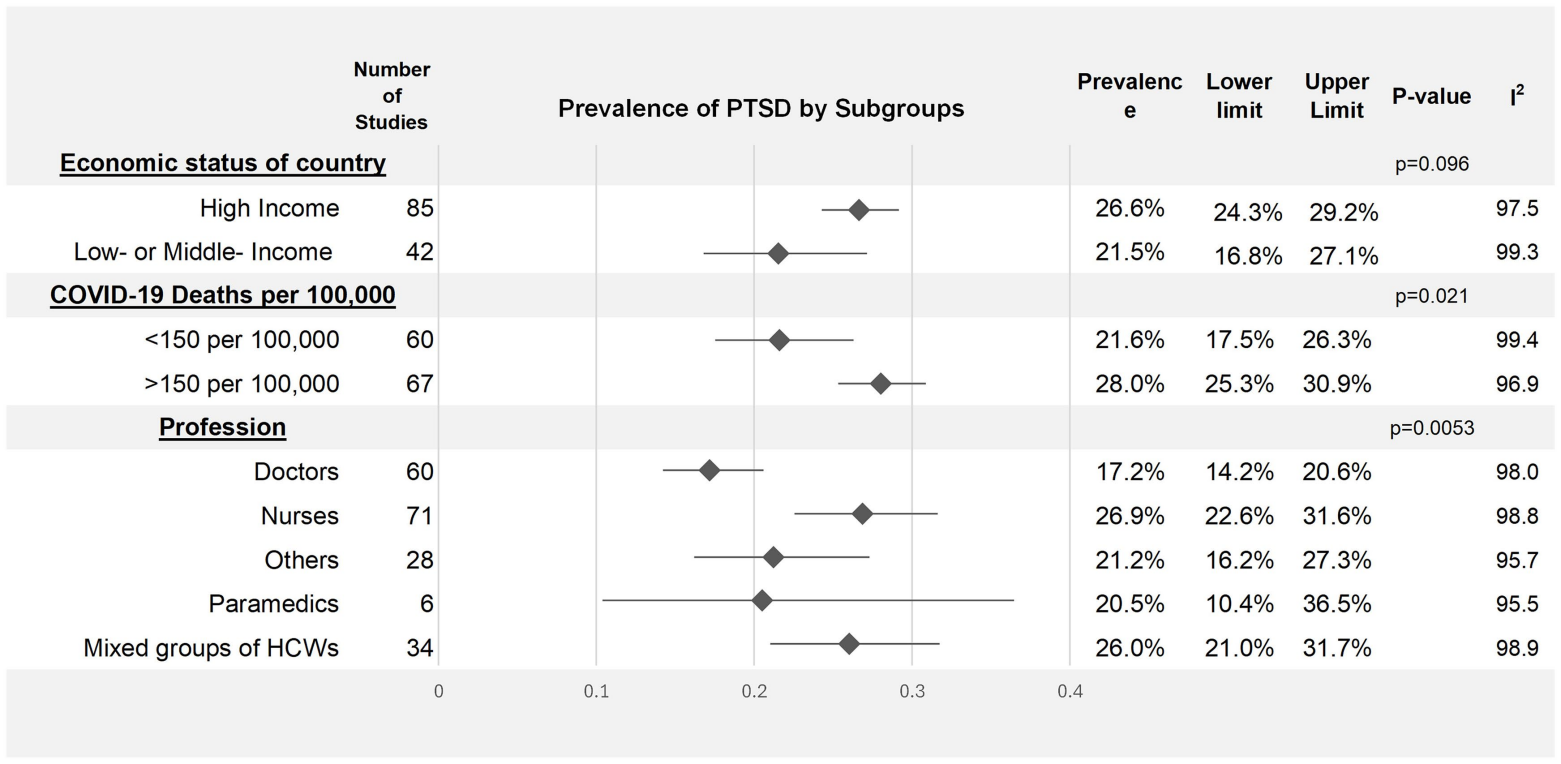
Forest Plot visualizing prevalence of probable PTSD among subgroups during COVID-19. PTSD denotes post-traumatic stress disorder.

Healthcare workers in high-income countries showed marginally higher estimated rates of probable PTSD (26.6, 95% CI: 24.3–29.2%) during the pandemic compared to low- and middle-income countries (21.5, 95% CI: 16.8–27.1%), however, this difference did not reach statistical significance (*p* = 0.096). Studies conducted in countries with higher COVID-19 mortality showed significantly higher rates of PTSD amongst HCWs (28.0, 95% CI: 25.3–30.9%) compared to countries with lower mortality (21.6, 95% CI 17.5–26.3%, *p* = 0.021) (Figure 4).

Across all studies, the most frequently used PTSD screening instruments were PCL-based tools (63 studies using PCL-C, PCL-5 or abbreviated PCL tools), followed by IES-based tools (51 studies using IES-R or abbreviated versions), and PC-PTSD tools (15 studies using PC-PTSD or PC-PTSD-5). The remaining 19 studies used other tools including TSQ, ITQ, DTS, and PDS-5. Significant differences were observed in the estimated pooled prevalence of probable PTSD based on the type of instrument used, both prior to COVID-19 (*p* = 0.047) and during COVID-19 (*p* = 0.016). However, the direction of these differences varied across the two time periods (see Supplementary Figure 1).

Of the 21 studies published in the 3 years prior to the pandemic, one study (4.8%) scored from 0 to 3 and was considered low quality, 15 studies (71.4%) scored from 4 to 6 and were considered fair quality, and five studies (23.8%) scored 7–9 and were considered high quality. The most common risks of bias in these studies were inadequate or unjustified sample size (18 studies or 85.7%), no acknowledgement of, or poor representation of identified target population (17 studies or 81.0%) and inadequate or unknown response rate (16 studies or 76.2%).

Of the 129 studies reporting data collected during the COVID-19 pandemic, 4 studies (3.1%) were of low quality, 83 studies (64.3%) of fair quality, and 42 studies (32.6%) of high quality (see Supplementary Table 2). Amongst these 129 studies, risks of bias included unrepresentative sample of target population (111 studies or 86.0%), unjustified or inadequate sample size (*n* = 108; 83.7%) and unknown or low response rates (*n* = 91; 70.5%).

Between-group analyses demonstrated a significantly higher prevalence rate among low quality studies compared to high quality studies both prior to COVID-19 (*p* = 0.0014) and during COVID-19 (*p* = 0.010; see Supplementary Figure 2). However, sensitivity analyses demonstrated that the removal of low quality studies did not substantially change the overall pooled prevalence estimate (prior to COVID-19: 16.3% vs. 15.5%; during COVID-19: 24.2% vs. 24.8%). The prevalence estimates of fair quality studies were similar to those of high quality studies prior to COVID-19 (*p* = 0.33), however they were significantly higher during COVID-19 (*p* = 0.014). A between-group analysis of studies with both a defined sampling frame and adequate response rate vs. those without found no significant difference in prevalence estimates either prior to COVID-19 (*p* = 0.57) or during COVID-19 (*p* = 0.11) (see Supplementary Figure 3).

Of all studies published in the 3 years prior to COVID-19, only 13 of 21 studies specified the year of data collection, ranging from 2012 until 2018. A meta-regression of year of data collection was conducted to assess any secular trend bias, and no significant trends in prevalence were observed over these years (*β* = −0.07, 95%CI: −0.27 to 0.13, *p* = 0.48, see Supplementary Figure 4).

Sensitivity analyses using the one-study-removed approach found no significant differences in the estimated mean prevalence, suggesting no single study had an inordinate impact on overall prevalence estimates, either prior to, or during, COVID-19 (see Supplementary Figures 5a,b).

Visualization of funnel plots (shown in Supplementary Figure 6) revealed no apparent asymmetry in studies published prior to COVID-19, whilst slight asymmetry can be seen in the studies during COVID-19. This was confirmed using Egger’s tests which showed no significant indication of publication bias in studies prior to COVID-19 (*p* = 0.28), whilst there was a trend towards a small study effect amongst studies during COVID-19, but this did not reach statistical significance (*p* = 0.054). High heterogeneity was reported among all subgroups (*I*^2^ range: 82.3–99.4%).

## 4 Discussion

This systematic review and meta-analysis provides the most comprehensive summary of studies to date assessing the prevalence of probable PTSD in HCWs. Crucially, it is also the first to systematically examine the impact of the COVID-19 pandemic on the global prevalence of probable PTSD among this workforce. Our results suggest that working through the COVID-19 pandemic had a substantial impact on the mental health of HCWs, with probable PTSD prevalence estimations increasing from 16% pre-pandemic to 25% during the pandemic. This effect was observed globally, although our results demonstrate a greater impact in countries with higher COVID-19 mortality. Importantly, estimated PTSD rates returned to pre-pandemic levels by 2022, suggesting this psychological impact was largely temporary. While this is a reassuring finding, it should be noted that the ‘baseline’ rate of PTSD found in this meta-analysis is substantially higher than that observed in the general population and higher than that reported amongst other trauma exposed workforces, including first responders and military personnel (169, 170). These findings suggest that the issue of PTSD amongst HCWs requires close attention.

Our finding of a 24.8% pooled prevalence of probable PTSD during COVID-19 is not far from estimates of 20.9% (15) and 21.5% (13, 14) reported in previous systematic reviews. These rates also align with prevalence estimates of PTSD in HCWs pooled across multiple viral epidemics (10, 171), indicating that the mental health consequences observed are not unique to the COVID-19 pandemic. The rise in rates of positive PTSD screening observed after the COVID-19 outbreak can likely be explained by increased exposure to potentially traumatic situations along with numerous additional psychosocial hazards cited by HCWs in qualitative evidence, including increased work hours, immense structural changes in the workplace, social isolation, moral injury due to insufficient resources and risk of infection (6, 8). It is important to note that all studies except one (101) utilized screening tools rather than clinical PTSD diagnoses. Screening tools can overestimate the prevalence of mental health disorders (172) and may be more sensitive to short-term distress rather than, necessarily, highlighting a need for clinical care, particularly when employed in an occupational context (173). As such, prevalence rates reported in the current review should be interpreted with caution. Nonetheless, a key strength of this review lies in the pre-pandemic comparison, and time trends in prevalence data which should remain valid.

Insights into the time trends of probable PTSD amongst HCWs during the course of the COVID-19 pandemic found the highest overall prevalence of probable PTSD was observed at the beginning of the pandemic in 2020, with a slight decrease in 2021 followed by a more significant decline in 2022. Contextually, COVID-19 deaths peaked in January 2021 and steadily declined throughout 2021 and 2022 (5). Declining prevalence of probable PTSD may reflect reduced burden on healthcare systems over time. Moreover, the distribution of COVID-19 vaccinations commenced in January 2021, which has been associated with better mental health outcomes among HCWs (174). However, only five studies with data collected in 2022 were included in the analysis, which may limit representativeness of the broader healthcare workforce. Further, the reduction in estimated prevalence may be partially attributable to workforce attrition, as HCWs with high levels of distress may have been more likely to exit the profession.

In accordance with previous literature (9, 11), subgroup analyses identified the highest rates of PTSD amongst nurses. This may be a reflection of nurses having closer contact with patients, inadequate support and training around trauma within the nursing profession, or a greater burden of other psychosocial workplace stressors. Qualitative evidence supports these theories, with nurses reporting prolonged patient contact compared to other professions, alongside chronic staffing shortages, unsafe work environments, overwhelming workloads, and lack of managerial support (175, 176). Nurses describe moral distress resulting from an inability to provide adequate patient care whilst adhering to COVID-19 protocols, as well as an erosion of work-life boundaries. Many nurses reported feeling unable to meet obligations to their own families due to occupational pressures, compounded by persistent fears of transmitting the virus to their families. Resilience training, education around trauma (3, 177), and collegial and managerial support have consistently been found to be protective factors within healthcare (3, 178, 179). Thus, implementing interventions addressing these factors for HCWs to support their own and their colleagues’ mental health may mitigate the risk of PTSD.

The estimated pooled prevalence of probable PTSD among HCWs in high-income countries was marginally higher than their counterparts in low- to middle-income countries. However, this finding did not reach statistical significance, and may have been driven by several confounders, such as cultural differences in participants’ willingness to disclose mental illness, varying levels of mental health literacy, or individual factors across settings (180). Additionally, this may reflect that more studies were undertaken in high-income areas disproportionately impacted by COVID-19, such as the USA and Italy, which had notably high infection and death rates (5). This theory is supported by our finding of significantly higher probable PTSD prevalence in countries with higher COVID-19 death rates. Although no causal relationship can be assumed, this suggests an association between the level of demand on health resources and probable PTSD prevalence amongst HCWs.

Mental ill-health among HCWs has been associated with impaired productivity, increased risk of medical errors, and overall lower quality of patient care (181). Therefore, implementing workplace initiatives to improve the wellbeing of HCWs is imperative not only for their personal health, but also for the broader impact on healthcare services. Many risk factors cited in both quantitative and qualitative research are modifiable (6, 8), and whilst some trauma exposure may be inevitable within healthcare, action must be taken by organizations and policy-makers to mitigate psychosocial hazards within the healthcare profession.

This study has several potential limitations that must be considered. Firstly, as previously discussed, most studies relied on self-report measures for assessing PTSD. Relevant to the current context, Scott and colleagues estimated the prevalence of PTSD in 11,000 hospital workers, using a self-report screening tool followed by a gold-standard diagnostic interview for a subsample of participants (172). This study found that the screening tool substantially overestimated the prevalence of PTSD. Consequently, the prevalence of PTSD, both prior to and during COVID-19 amongst this cohort may be overestimated in the present review. Additionally, there was substantial variation in the screening tools used to assess PTSD, using diagnostic criteria from both the DSM and ICD. One key strength of this review lies in its inclusion of only pre-established cut-off scores for each tool, ensuring high diagnostic accuracy, rather than relying on authors’ definitions of a positive screening for PTSD, which show major inconsistencies. Nevertheless, as studies across both periods used screening tools, the relative increase in post-traumatic stress symptomatology should remain valid.

Secondly, many studies used a non-defined sampling frame, such as social media or snowball recruitment, and few studies reported an adequate response rate. Particularly, the urgency to publish data during COVID-19 may have resulted in compromised methodological rigor (182). These biases are addressed in the JBI quality assessment tool, however, they may have an inordinate impact on the true risk of bias. Although our between-groups analyses found no significant difference in prevalence between studies with and without a defined sampling frame and adequate response rate, the risk of selection bias remains, and the reported estimated prevalence of probable PTSD should be interpreted with caution. During the COVID-19 period, studies of high quality yielded significantly lower prevalence estimates than studies of low quality, as such, the magnitude of increased PTSD symptomatology may be inflated in this review. Another limitation to consider is that literature searches were conducted based on publication date rather than the date of data collection, potentially missing studies conducted in the target periods. Although studies during COVID-19 were selected only if data collection was during the pandemic, many pre-pandemic studies collected data earlier than the specified publication period. To account for potential secular trend bias, a meta-regression examining prevalence over time was conducted and found no evidence of increasing prevalence rates in the lead up to the pandemic. Nonetheless, the pre-pandemic prevalence estimates reported in the present review may not accurately reflect the years immediately preceding the pandemic.

Potential cross-cultural limitations must be considered, as many of the PTSD screening tools were developed in Western contexts and may not accurately capture trauma responses across cultures (183). Additionally, only studies with an English language translation available were included, which may have further reduced global representation. Lastly, significant heterogeneity persisted in estimates of PTSD prevalence, despite performing several subgroup analyses. Whilst some studies measured PTSD symptomatology in response to any stressor identified by participants, many studies measured symptoms specifically in response to pandemic-related traumatic experiences, likely resulting in major inconsistencies in the outcomes being measured and driving further heterogeneity. High heterogeneity may also reflect other methodological differences, varying impacts of COVID-19 on healthcare systems, or differences in workplace-level factors correlated with PTSD, such as social support (3, 178, 179), mental health training (177), or access to resources or PPE (3, 9, 11).

## 5 Conclusion

In conclusion, this systematic review and meta-analysis has demonstrated concerningly elevated rates of probable PTSD in healthcare workers prior to the pandemic, which was significantly exacerbated during the first 2 years of the COVID-19 pandemic but appears to have returned to pre-pandemic levels by 2022. Nurses and HCWs in regions of higher COVID-19 mortality rates were found to be at increased risk. These findings highlight the need for the implementation of targeted, evidence-based preventative strategies and policies to mitigate the risk of PTSD in healthcare professionals at both the organizational and individual level. Particularly, early interventions should be established and readily accessible to better protect HCWs during and following healthcare emergencies.

## Data Availability

The original contributions presented in the study are included in the article/Supplementary material, further inquiries can be directed to the corresponding author.
