# Supplementary material for: Prevalence of post-traumatic stress disorder in healthcare workers before and during COVID-19: a systematic review and meta-analysis
Source: Front Public Health. 2026 Mar 6;14:1735552. doi: 10.3389/fpubh.2026.1735552 (PMC13003540; doi:10.3389/fpubh.2026.1735552)
Supplement: Supplementary file 1 [file Data_Sheet_1.docx]

Supplementary Material

Prevalence of Post-Traumatic Stress Disorder in Healthcare Workers Before and During COVID-19: A Systematic Review and Meta-Analysis

Contents

[1 Appendix 1. Search Strategy for studies 2](#_Toc216786582)

[2 Appendix 2. Exclusion Criteria and Data Extracted for Studies 5](#_Toc216786583)

[3 Table 1. Accepted PTSD Scales and Cut-off Scores 6](#_Toc216786584)

[4 Table 2. Inter-rater reliability across screening processes 7](#_Toc216786585)

[5 Table 3. Joanna Briggs Institute (JBI) Quality Assessment Ratings for Studies 8](#_Toc216786586)

[6 Figure 1. Pooled prevalence estimates across PTSD screening tools 14](#_Toc216786587)

[7 Figure 2. Pooled prevalence estimates for low, fair and high quality studies prior to and during COVID-19 15](#_Toc216786588)

[8 Figure 3. Pooled prevalence estimates among studies with an adequate sampling strategy and adequate response rate vs those without 16](#_Toc216786589)

[9 Figure 4. Meta-regression of prevalence over year prior to COVID-19 17](#_Toc216786590)

[10 Figure 5a. One-Study Removed Forest Plot for Studies Prior to COVID-19 18](#_Toc216786591)

[11 Figure 5b. One-Study Removed Forest Plot for Studies During COVID-19 19](#_Toc216786592)

[12 Figure 6. Funnel Plots Visualizing Possible Publication Bias 21](#_Toc216786593)

[13 References 22](#_Toc216786594)

# Appendix 1. Search Strategy for studies

Searches were not restricted by location or language, but only studies available with an English language translation available were selected. To identify articles that reported the prevalence of PTSD among health care workers prior to COVID-19, a search of PsycInfo, Embase, Cochrane, and PubMed was conducted on the 29th of November 2022. A restriction or filter of “January 2017” to “December 2019” was placed on the searches to capture studies only within the three-year period prior to COVID-19. Given that COVID-19 had broken out by December 2019, any studies identified in this search that were published during December 2019 were manually excluded unless explicitly stating the data collection period was prior to December 2019. Additionally, the first 10 pages of Google Scholar were searched using the generic terms of “Prevalence”, “Health Care Workers”, “PTSD”. To identity articles that reported the prevalence of PTSD among health care workers during COVID-19, a search of PsycInfo, Embase, Cochrane, PubMed, and ProQuest Coronavirus Research Database was conducted on 10th July 2023 with a restriction of filter of “from December 2019”. Additionally, the first 10 pages of Google Scholar were searched using the generic terms of “Prevalence”, “Health Care Workers”, “PTSD”, “COVID-19”.

|  | **Population: Health Care Workers** | | **Outcome: PTSD** | | **Context: COVID (only when searching for studies conducted during COVID-19)** | | **Study Design: Prevalence Studies** | |
| --- | --- | --- | --- | --- | --- | --- | --- | --- |
| **Database** | **Subject Headings** | **Keywords** | **Subject Headings** | **Keywords** | **Subject Headings** | **Keywords** | **Subject Headings** | **Keywords** |
| **Free Text** |  | health personnel OR  health care personnel OR healthcare personnel OR health care work* OR  healthcare work* OR health work* OR  health professional* OR  health care professional* OR healthcare professional* OR medical care personnel OR medical personnel OR  medical staff OR medical professional* OR nurs* OR physician* OR doctor* OR paramedic* OR |  | posttraumatic stress disorder* OR PTSD OR post-traumatic stress disorder* |  | coronavirus OR "coronavirus" OR coronavirinae OR coronaviridae OR betacoronavirus OR covid19 OR "covid 19" OR "COVID-19" OR nCoV OR "CoV 2" OR CoV2 OR sarscov2 OR 2019nCoV OR "novel CoV" OR "wuhan virus" OR "severe acute respiratory syndrome*" OR 19nCoV* OR "2019 novel*" OR "SARS-CoV-2*" OR "SARSCoV-2*" OR SARSCoV2* OR "SARSCoV2*" OR "COVID*2” OR "Coronavirus infections" |  | cohort analysis or epidemiology or prospective study or retrospective study or Prevalence Study |
| **Embase (Ovid)** | Health care personnel/ or health workforce/ or exp hospital personnel/ or exp medical personnel/ or exp paramedical  personnel/ | b l  (health personnel  OR  health care personnel OR healthcare personnel OR health care work* OR  healthcare work* OR health work* OR  health professional* OR  health care professional* OR healthcare professional* OR medical care personnel OR medical personnel OR  medical staff OR medical professional* OR nurs* OR physician* OR doctor* OR | posttraumatic stress disorder/ | (posttraumatic stress disorder* OR PTSD OR posttraumatic stress disorder*).tw | exp coronavirus disease 2019/ OR exp Severe acute  respiratory syndrome coronavirus 2/ | (coronavirus OR "coronavirus" OR coronavirinae OR coronaviridae OR betacoronavirus OR covid19 OR "covid 19" OR "COVID-19" OR nCoV OR "CoV 2" OR CoV2 OR sarscov2 OR 2019nCoV OR "novel CoV" OR "wuhan virus" OR "severe acute respiratory syndrome*" OR 19nCoV* OR "2019 novel*" OR "SARS-CoV-2*" OR "SARSCoV-2*" OR SARSCoV2* OR "SARSCoV2*" OR "COVID*2" OR “Coronavirus infections").tw | exp prevalence/ OR cohort analysis/ OR exp epidemiology/ OR prospective study/ OR retrospective study/ | (cohort analysis or epidemiology or prospective study or retrospective study or Prevalence Study).tw. |
| **PubMed** | "Health  Personnel"[Mesh] | di * OR  health personnel[tw] OR health care personnel[tw] OR healthcare personnel[tw] OR health care work*[tw] OR healthcare work*[tw] OR health work*[tw] OR health professional*[tw] OR health care professional*[tw] OR healthcare professional*[tw] OR medical care personnel[tw] OR medical personnel[tw] OR medical staff[tw] OR medical professional*[tw] OR nurs*[tw] OR physician*[tw] OR doctor*[tw] OR paramedic*[tw] OR | "Stress Disorders,  Post-  Traumatic"[Mesh] | Stress Disorders[tw]  OR Post-Traumatic  Trauma[tw] OR  Stressor Related  Disorders [tw] OR  Psychological Trauma [tw] OR posttraumatic stress disorder*[tw] PTSD [tw] OR posttraumatic stress disorder [tw] | COVID-19[Mesh] OR  "SARS-CoV2"[Mesh] OR Betacoronavirus"[M eSH Terms] | coronavirus[tw] OR "coronavirus"[tw] OR coronavirinae[tw] OR coronaviridae[tw] OR betacoronavirus[tw] OR covid19[tw] OR "covid 19"[tw] OR nCoV[tw] OR "CoV 2"[tw] OR CoV2[tw] OR sarscov2[tw] OR 2019nCoV[tw] OR  "novel CoV"[tw] OR  "wuhan virus"[tw] OR  "severe acute respiratory syndrome*"[tw] OR 19nCoV*[tw] OR  "2019 novel*"[tw] OR  "SARS-CoV-2*"[tw] OR  "SARSCoV-2*"[tw] OR  SARSCoV2*[tw] OR  "SARS-CoV2*"[tw] | (("Prevalence"[Mesh] OR  "Epidemiology"[Mesh] OR  "epidemiology"  [Subheading] OR “CrossSectional Studies"[Mesh])  OR (“Longitudinal  Studies"[Mesh] OR  "Prospective  Studies"[Mesh])) OR  "Retrospective  Studies"[Mesh] |  |
| **ProQuest Coronavirus Research Database** |  | b l  Abstract,Title(health personnel OR health care personnel OR healthcare personnel OR health care work* OR healthcare work* OR health work* OR health professional* OR health care professional* OR healthcare professional* OR medical care personnel OR medical personnel OR medical staff OR medical professional* OR nurs* OR physician* OR doctor* OR paramedic* OR ambulance personnel OR emergency medical technician*) |  | Abstract,Title(Stress  Disorders OR PostTraumatic Trauma  OR Stressor Related  Disorders OR  Psychological Trauma OR posttraumatic stress disorder* OR PTSD OR post-traumatic stress disorder*) |  |  | Abstract,Title(cohort OR cross-sectional OR epidemiol* OR incidence OR occurrence OR proportion OR rate OR probability OR longitudinal OR observational OR population OR prevalence OR prospective OR retrospective) |  |
| **Cochrane** | Health Personnel [MESH] | health personnel  OR  "health care personnel" OR "healthcare personnel" OR  "health care work*"  OR  "healthcare work*"  OR  "health work*" OR  "health  professional*" OR "health care professional*" OR "healthcare professional*" OR "medical care personnel" OR  "medical personnel"  OR  "medical staff" OR  "medical  professional*" OR "nurs*" OR  "physician*" OR  "doctor*" OR  "paramedic*" OR | Stress Disorders, Post | "Stress Disorders"  OR  "Post-Traumatic  Trauma" OR  "Stressor Related  Disorders" OR  "Psychological  Trauma" OR "posttraumatic stress disorder*" OR  "PTSD" OR "post-traumatic stress disorder*" | Coronavirus (Mesh)  OR  Coronavirus  Infections (Mesh)  OR  COVID-19 (Mesh) OR  SARS-CoV-2 (Mesh)  OR  Betacoronavirus  (Mesh) | """coronavirus"" OR  “corona virus” OR  ""coronavirinae"" OR  ""coronaviridae"" OR  ""betacoronavirus"" OR  ""covid19"" OR  “covid 19” OR  ""nCoV"" OR  “CoV 2” OR  ""CoV2"" OR  ""sarscov2"" OR  ""2019nCoV"" OR  “novel CoV” OR  “wuhan virus” OR  ""severe acute respiratory syndrome*"" OR  ""19nCoV*"" OR  ""2019 novel*"" OR  ""SARS-CoV-2*"" OR  ""SARSCoV-2*"" OR  ""SARSCoV2*"" OR  ""SARS-CoV2*"" OR  ""COVID*2" |  |  |
| **PsycInfo** |  | Abstract,Title(health personnel OR health care personnel OR healthcare personnel OR health care work* OR healthcare work* OR health work* OR health professional* OR health care professional* OR healthcare professional* OR medical care personnel OR medical personnel OR medical staff OR medical professional* OR nurs* OR physician* OR doctor* OR paramedic* OR ambulance personnel OR emergency medical technician*) |  | Abstract,Title(Stress  Disorders OR PostTraumatic Trauma  OR Stressor Related  Disorders OR  Psychological Trauma OR posttraumatic stress disorder* OR PTSD OR post-traumatic stress disorder*) |  | noft(coronavirus OR "coronavirus" OR coronavirinae OR coronaviridae OR betacoronavirus OR covid19 OR "covid 19" OR "COVID-19" OR nCoV OR "CoV 2" OR CoV2 OR sarscov2 OR 2019nCoV OR "novel CoV" OR "wuhan virus" OR "severe acute respiratory syndrome*" OR 19nCoV* OR "2019 novel*" OR "SARS-CoV-2*" OR "SARSCoV-2*" OR SARSCoV2* OR "SARSCoV2*" OR "COVID*2" OR"Coronavirus infections") |  | Abstract,Title(cohort  OR cross-sectional OR epidemiol* OR incidence OR occurrence OR proportion OR rate OR probability OR longitudinal OR observational OR population OR prevalence OR prospective OR retrospective) |

# Appendix 2. Exclusion Criteria and Data Extracted for Studies

Papers were excluded if they:

1. Were not available in English;
2. Were of incorrect format (e.g. systematic reviews, trials) or were not peer-reviewed (e.g. conference abstracts, dissertations);
3. Included HCWs that were not working during the pandemic (e.g. retired or on leave) or HCWs that were not in primarily patient-facing, clinical roles (e.g. administrative or cleaning personnel);
4. Were based in a war-zone;
5. Included HCWs that were only screened for PTSD if they fulfilled a certain criteria (e.g. diagnosed with COVID-19) or had experienced a certain event (e.g. violence, trauma);
6. Did not use a validated tool to screen for PTSD, or the screening tool was based off a version of the Diagnostic and Statistical Manual of Mental Disorders (DSM) earlier than the third edition-revised (DSM-III-R) or equivalent International Classification of Diseases (ICD);
7. Did not report the cut-off score, or used a cut-off score deemed outside of acceptable by the review team;
8. The data was reported in another study, or participants overlapped with another study.

In instances where the same participants were used in multiple papers, the most comprehensive paper was included. This was determined based on sample size, or where participant demographics were described more thoroughly. In instances where longitudinal data were reported within the same year, baseline data only were extracted. In instances where data were reported longitudinally across multiple years of COVID-19, data from the earliest collection point in each year were extracted and used for only by-year analysis.

The following information was extracted from each included study according to the protocol:

1. Study ID (Author, Year);
2. Study title;
3. DOI;
4. Date of Publication;
5. Journal Name;
6. Study type (cross-sectional or longitudinal);
7. Country and study setting;
8. Dates of data collection;
9. Profession type of participants;
10. Demographic details of participants;
11. Recruitment details and sample size;
12. PTSD scale/instrument and cut-off score;
13. Response rate to scale;
14. PTSD prevalence data (raw number of participants screening above cut-off for PTSD over raw number of participants screened).

# Table 1. Accepted PTSD Scales and Cut-off Scores

Table 1. Cut-off scores, sensitivity, and specificity for PTSD screening tools accepted in the review. Abbreviations: DSM-5, Diagnostic and Statistical Manual of Mental Disorders 5; DTS, Davidson Trauma Scale; DTS-8, 8-item DTS; IES-R, Impact of Event Scale – Revised; IES-6, 6-item Impact of Event Scale; ITQ, International Trauma Questionnaire; PCL, PTSD Checklist; PCL4-5, 4-item PCL-5; PCL-5, PCL for DSM-5; PCL-6, 6-item PCL; PCL-C, PCL-Civilian; PC-PTSD, Primary Care-PTSD Screen; PC-PTSD-5, PC-PTSD for DSM-5; PDS-5, Post-traumatic Diagnostic Scale for DSM-5; PSS-SR, PTSD Symptom Scale – Self-Report Version; PTSD, Post-traumatic stress disorder; PTSS-10, 10-item Post-traumatic Stress Scale; PTSS-14, 14-item Post-traumatic Stress Scale; SCID-5, Structural Clinical Interview for DSM-5 Disorders; TSQ, Trauma Screening Questionnaire.

| **PTSD Scale** | **Accepted cut-off scores** | **Sensitivity** | **Specificity** | **Reference** |
| --- | --- | --- | --- | --- |
| Davidson Trauma Questionnaire | 40 | 0.69 | 0.95 | (1) |
| 8-item short-version DTS | 12 | N/A  PPV: 1.0 | N/A  NPV: 0.89 | (2) |
| IES-R | 33-34 | 0.86  0.91 | 0.80  0.82 | For 34 (3)  For 33 (4) |
| IES-6 | 10 or 1.75 mean | 0.88 | 0.85 | For 1.75 mean (5) |
| ITQ | Symptom cluster method (>=2 in each subscale) | 0.71 | 0.91 | (6) |
| PCL (PCL-S and PCL-C) | 44-50 | 0.94-0.78 | 0.86-0.86 | (7) |
| PCL-5 | 31-33 | 0.88 | 0.69 | (8) |
| 8-item abbrv. PCL-5 | 13-14 | 0.96-0.90 | 0.90-0.92 | (9) |
| 4-item abbrv. PCL-5 | 8 | 0.81 | 0.94 | (9) |
| PC-PTSD | 3 | 0.78 | 0.87 | (10) |
| PC-PTSD-5 | 3 | 0.95 | 0.85 | (11) |
| PDS-5 | 28 | 0.79 | 0.78 | (12) |
| PTSS-14 | 45 | 0.86 | 0.97 | (13) |
| PTSD-8 | 3 in each subscale | 0.71-0.80 | 0.95-0.88 | (14) |
| PCL-6 | 14 | 0.80 | 0.76 | (15) |
| PSS-SR | 14 | 0.83 | 0.71 | (16) |
| TSQ | 6 | 0.86 | 0.93 | (17) |
| Short Screening Scale for DSM-IV-PTSD (Breslau) | 4 | 0.80 | 0.97 | (18) |

# Table 2. Inter-rater reliability across screening processes

|  |  | **Prior to COVID-19**  **Cohen’s κ** | | **During COVID-19**  **Cohen’s κ** | |
| --- | --- | --- | --- | --- | --- |
| **Reviewer pairs** | | **Title and abstract screening** | **Full text screening** | **Title and abstract screening** | **Full text screening** |
| KK | EG | 0.72 | No screening | 0.78 | 0.93 |
| KK | VLD | 0.77 | No screening | No screening | No screening |
| CF | DC | No screening | 0.68 | No screening | 0.67 |
| KK | NK | No screening | No screening | 0.70 | 0.90 |
| KK | DC | No screening | No screening | 0.84 | 0.88 |

Table 2. Inter-rater reliability across reviewer pairs during title and abstract screening and full-text screening as measured by Cohen’s κ.

# Table 3. Joanna Briggs Institute (JBI) Quality Assessment Ratings for Studies

| **Study ID** | **Q1** | **Q2** | **Q3** | **Q4** | **Q5** | **Q6** | **Q7** | **Q8** | **Q9** | **Total QA Score** |
| --- | --- | --- | --- | --- | --- | --- | --- | --- | --- | --- |
| Prior to COVID-19 | | | | | | | | | | |
| Carleton 2018 | 1 | 0 | ? | 0 | ? | 1 | 1 | ? | ? | 3 |
| Cohen 2017 | 1 | 1 | ? | 0 | ? | 1 | 1 | 1 | 0 | 5 |
| Colville 2017 | 1 | 1 | ? | 1 | ? | 1 | 1 | 1 | 1 | 7 |
| Eiche 2019 | 1 | 1 | ? | 1 | 1 | 1 | 1 | ? | 0 | 6 |
| Hilton 2017 | 1 | 1 | ? | 0 | ? | 1 | 1 | 1 | 0 | 5 |
| Jackson 2019 | 1 | 1 | ? | 1 | ? | 1 | 1 | 1 | 0 | 6 |
| Jackson 2019 | 1 | 1 | ? | 1 | ? | 1 | 1 | 1 | 0 | 6 |
| Kannan 2019 | 1 | 1 | ? | 0 | ? | 1 | 1 | 1 | 1 | 6 |
| Kerai 2017 | 1 | 1 | 1 | 1 | 0 | 1 | ? | 1 | 1 | 7 |
| Khazaei 2019 | 1 | 1 | 1 | 1 | ? | 1 | 1 | 1 | 1 | 8 |
| Kucmin 2018 | 1 | 1 | ? | 1 | 1 | 1 | ? | ? | 0 | 5 |
| Leinweber 2017 | 1 | 1 | 1 | 1 | ? | 1 | 1 | 1 | 0 | 7 |
| Linane 2019 | 1 | 1 | ? | 0 | ? | 1 | 1 | 1 | 0 | 5 |
| Luftman 2017 | 1 | 1 | ? | 0 | ? | 1 | 1 | ? | ? | 4 |
| McFarland 2017 | 1 | 1 | ? | 1 | 1 | 1 | 1 | ? | 0 | 6 |
| Rodriquez-Rey 2019 | 1 | 1 | ? | 1 | ? | 1 | 1 | ? | 0 | 5 |
| Rybojad 2019 | 1 | 1 | ? | 1 | ? | 1 | 1 | 1 | 0 | 6 |
| Schafer 2018 | 1 | 1 | ? | 1 | ? | 1 | 1 | 1 | 0 | 6 |
| Thompson 2017 | 1 | 0 | ? | 1 | ? | 1 | 1 | 1 | ? | 5 |
| van Steijn 2019 | 1 | 1 | ? | 1 | 1 | 1 | 0 | 1 | 0 | 6 |
| Zhou 2018 | 1 | 1 | 0 | 1 | ? | 1 | 1 | 1 | 1 | 7 |
| During COVID-19 | | | | | | | | | | |
| Amsalem 2021 | 1 | 0 | ? | 1 | 0 | 1 | 1 | 1 | ? | 5 |
| Arnetz 2020 | 1 | 0 | 1 | 1 | 1 | 1 | 1 | 1 | ? | 7 |
| Bani Issa 2022 | 1 | 1 | 0 | 1 | ? | 1 | 1 | 1 | 1 | 7 |
| Bassi 2021 | 1 | 0 | ? | 1 | ? | 1 | 1 | ? | ? | 4 |
| Bates 2021 | 1 | 1 | ? | 1 | 1 | 1 | 1 | 1 | 1 | 8 |
| Baumann 2021 | 1 | 1 | ? | 1 | 1 | 1 | 1 | 1 | 1 | 8 |
| Blanco-Daza 2022 | 1 | 1 | 1 | 1 | ? | 1 | 1 | 1 | 0 | 7 |
| Bonzini 2022 | 1 | 1 | ? | 1 | ? | 1 | 1 | 1 | 1 | 7 |
| Caillet 2020 | 1 | 1 | ? | 1 | 1 | 1 | 1 | 1 | 1 | 8 |
| Carola 2022 | 1 | ? | 0 | 0 | ? | 1 | 1 | ? | ? | 3 |
| Chang 2022 | 0 | 1 | ? | 0 | ? | 1 | 1 | 1 | 0 | 4 |
| Chatzittofis 2021 | 1 | 0 | ? | 1 | ? | 1 | 1 | 1 | ? | 5 |
| Chauhan 2021 | 1 | 1 | 1 | 1 | ? | 1 | ? | 1 | 0 | 6 |
| Che 2022 | 1 | 1 | 1 | 1 | ? | 1 | 1 | ? | 1 | 7 |
| Chen 2020 | 1 | 1 | ? | 1 | ? | 1 | 1 | 1 | 1 | 7 |
| Cobo-Cuenca 2022 | 1 | 1 | 1 | 1 | ? | 1 | 1 | 1 | 0 | 7 |
| Conti 2020 | 1 | 0 | ? | 1 | ? | 1 | 1 | 1 | ? | 5 |
| Costantini 2022 | 1 | 1 | ? | 1 | ? | 1 | 1 | 1 | 0 | 6 |
| Couper 2022 | 1 | 0 | ? | 1 | 1 | 1 | 1 | 1 | ? | 6 |
| Crowe 2021 | 1 | 1 | ? | 1 | ? | 1 | 1 | 1 | 0 | 6 |
| D'Alessandro-Lowe 2023 | 1 | 0 | ? | 1 | 0 | 1 | 1 | ? | ? | 4 |
| Damico 2022 | 1 | 1 | ? | 1 | ? | 1 | 1 | ? | 1 | 6 |
| Dehon 2021 | 1 | 1 | 1 | 1 | 1 | 1 | 1 | 1 | 1 | 9 |
| de Lima Osorio 2023 | 1 | 0 | ? | 0 | ? | 1 | 1 | ? | ? | 3 |
| Demartini 2020 | ? | 0 | ? | 0 | ? | 1 | 1 | 1 | ? | 3 |
| Di Tella 2020 | 1 | 0 | ? | 1 | ? | 1 | 1 | 1 | ? | 5 |
| Dykes 2022 | 1 | 1 | ? | 1 | ? | 1 | ? | 1 | 0 | 5 |
| Engelbrecht 2021 | 1 | 0 | ? | 1 | ? | 1 | 1 | 1 | ? | 5 |
| Fattori 2021 | 1 | 1 | ? | 1 | ? | 1 | 1 | 1 | 1 | 7 |
| Feingold 2021 | 1 | 1 | ? | 1 | ? | 1 | 1 | 1 | 1 | 7 |
| Fournier 2022 | 1 | 1 | ? | 1 | 1 | 1 | 1 | ? | ? | 6 |
| Gainer 2021 | 1 | 0 | ? | 1 | 0 | 1 | 1 | ? | ? | 4 |
| Geng 2021 | 1 | 1 | ? | 1 | ? | 1 | 1 | 1 | 1 | 7 |
| Ghio 2021 | 1 | 1 | ? | 1 | ? | 1 | 1 | 1 | 0 | 6 |
| Gonzalez Mendez 2022 | 1 | 0 | ? | 1 | ? | 1 | 1 | 1 | ? | 5 |
| Gonzalez-Mesa 2021 | 1 | 0 | ? | 1 | ? | 1 | 1 | ? | 0 | 4 |
| Gorini 2022 | 1 | 0 | ? | 1 | ? | 1 | 1 | 1 | ? | 5 |
| Greenberg 2021 | 1 | 1 | ? | 0 | ? | 1 | 1 | 1 | 0 | 5 |
| Guardiano 2022 | 1 | 0 | ? | 1 | ? | 1 | 1 | 1 | ? | 5 |
| Guo 2021 | 1 | 1 | ? | 1 | ? | 1 | 1 | 1 | 1 | 7 |
| Guttormson 2022 | 1 | 0 | ? | 1 | ? | 1 | 1 | 1 | ? | 5 |
| Habtamu 2021 | 1 | 1 | 0 | 1 | ? | 1 | 1 | 1 | 1 | 7 |
| Havaei 2021 | 1 | 1 | ? | 0 | ? | 1 | 1 | 1 | 1 | 6 |
| Heesakkers 2021 | 1 | 0 | ? | 1 | 1 | 1 | 1 | 1 | 0 | 6 |
| Heesakkers 2023 | 1 | 0 | ? | 1 | ? | 1 | 1 | 1 | 0 | 5 |
| Hendrickson 2022 | 1 | 0 | ? | 1 | ? | 1 | 1 | 1 | ? | 5 |
| Hernandez 2021 | 1 | 0 | ? | 0 | 0 | 1 | 1 | 1 | ? | 4 |
| Hickling 2022 | 1 | 0 | ? | 1 | ? | 1 | 1 | 1 | ? | 5 |
| Hill 2021 | 1 | 1 | ? | 0 | ? | 1 | 1 | 1 | 1 | 6 |
| Honarmand 2022 | 1 | 1 | 1 | 1 | ? | 1 | 1 | 1 | ? | 7 |
| Ibrahim 2023 | 1 | 1 | ? | 1 | ? | 1 | 1 | 1 | 0 | 6 |
| Iqbal 2021 | 1 | ? | 1 | 0 | ? | 1 | 1 | 1 | ? | 5 |
| James 2022 | 1 | 1 | ? | 1 | ? | 1 | 1 | 1 | 0 | 6 |
| Johns 2022 | 1 | 0 | 1 | 1 | 0 | 1 | 1 | 1 | ? | 6 |
| Jovarauskaite 2022 | 1 | 0 | 1 | 1 | ? | 1 | 1 | 1 | ? | 6 |
| Kabunga 2021 | 1 | 1 | 0 | 1 | ? | 1 | 1 | 1 | 1 | 7 |
| Kachadourian 2021 | 1 | 1 | ? | 1 | ? | 1 | 1 | 1 | 0 | 6 |
| Kader 2021 | 1 | 1 | ? | 1 | ? | 1 | 1 | 1 | 1 | 7 |
| Kalyanaraman 2022 | 1 | 1 | 1 | 1 | ? | 1 | 1 | 1 | 0 | 7 |
| Kim 2022 | 1 | 1 | ? | 1 | ? | 1 | 1 | 1 | ? | 6 |
| King 2022 | 1 | 1 | ? | 1 | ? | 1 | 1 | 1 | 0 | 6 |
| Korkut 2022 | 0 | 1 | ? | 1 | ? | 1 | ? | 1 | ? | 4 |
| Kumar 2023 | 1 | 1 | ? | 1 | ? | 1 | 1 | 1 | 1 | 7 |
| Kwobah 2021 | 1 | 0 | 0 | 1 | ? | 1 | 1 | 1 | ? | 5 |
| Lamb 2021 | 1 | 1 | ? | 1 | 1 | 1 | 1 | 1 | 0 | 7 |
| Laurent 2022 | 1 | 1 | ? | 1 | ? | 1 | 1 | ? | ? | 5 |
| Lee 2021 | 1 | ? | ? | 1 | ? | 1 | ? | 1 | 1 | 5 |
| Lee 2022 | 1 | 1 | ? | 1 | ? | 1 | 1 | 1 | 1 | 7 |
| Lei 2021 | ? | 0 | ? | 0 | ? | 1 | 1 | 1 | ? | 3 |
| Levi 2022 | 1 | 0 | 0 | 1 | 0 | 1 | 1 | 1 | ? | 5 |
| Li 2021 | 1 | 1 | ? | 1 | ? | 1 | 1 | 1 | 1 | 7 |
| Li 2022a | 1 | ? | ? | 1 | ? | 1 | 1 | 1 | 0 | 5 |
| Li 2022b | 1 | 1 | ? | 1 | ? | 1 | 1 | 1 | ? | 6 |
| Liu 2023 | 1 | 1 | ? | 1 | ? | 1 | 1 | 1 | ? | 6 |
| Lixia 2022 | 1 | 1 | ? | 1 | ? | 1 | 1 | 1 | 1 | 7 |
| Lombard 2022 | 1 | 1 | 1 | 1 | ? | 1 | 1 | 1 | 0 | 7 |
| Lopez-Salinas 2023 | 1 | 1 | ? | 1 | ? | 1 | 1 | 1 | 0 | 6 |
| Lu 2021 | 1 | 1 | ? | 1 | ? | 1 | 1 | ? | 0 | 5 |
| Marcomini 2021 | 1 | 1 | ? | 1 | ? | 1 | 1 | 1 | 1 | 7 |
| Marsden 2022 | 1 | 1 | ? | 1 | ? | 1 | 1 | 1 | 1 | 7 |
| Martinez-Caballero 2021 | 1 | 1 | ? | 1 | ? | 1 | 1 | 1 | 0 | 6 |
| Martsenkovskyi 2022 | 1 | 0 | 1 | 1 | ? | 1 | 1 | 1 | ? | 6 |
| McGuinness 2022 | 1 | 1 | ? | 1 | 1 | 1 | 1 | 1 | 0 | 7 |
| McGuinness 2023 | 1 | 1 | ? | 1 | 1 | 1 | 1 | ? | 0 | 6 |
| Mehta 2022 | 1 | 1 | ? | 1 | ? | 1 | 1 | 1 | ? | 6 |
| Minelli 2022 | 1 | 0 | ? | 1 | ? | 1 | 1 | 1 | ? | 5 |
| Mohsin 2022 | ? | 1 | 0 | 1 | ? | 1 | 1 | 1 | 0 | 5 |
| Mosheva 2021 | 1 | 1 | ? | 1 | 1 | 1 | 1 | 1 | 0 | 7 |
| Murata 2021 | 1 | 0 | ? | 1 | ? | 1 | 1 | ? | ? | 4 |
| Ng 2022a | 1 | 0 | ? | 1 | ? | 1 | 1 | 1 | ? | 5 |
| Ng 2022b | 1 | 1 | ? | 1 | ? | 1 | 1 | 1 | 0 | 6 |
| Ouazzani Housni Touhami 2022 | 1 | ? | ? | 1 | ? | 1 | 1 | 1 | 1 | 6 |
| Ouyang 2022 | 1 | 1 | 1 | 1 | 1 | 1 | 1 | ? | 1 | 8 |
| Oz Tuncer 2022 | 1 | 1 | ? | 1 | ? | 1 | 1 | 1 | 1 | 7 |
| Pan 2021 | 1 | 0 | 1 | 1 | ? | 1 | 1 | 1 | ? | 6 |
| Pappa 2021 | 1 | 1 | ? | 1 | ? | 1 | 1 | 1 | 0 | 6 |
| Pascoe 2022a | 1 | 0 | ? | 1 | 1 | 1 | 1 | ? | ? | 5 |
| Pascoe 2022b | 1 | 0 | ? | 1 | 0 | 1 | 1 | 1 | ? | 5 |
| Pasin 2020 | 1 | ? | ? | 1 | ? | 1 | 1 | ? | 0 | 4 |
| Qureshi 2020 | 1 | ? | ? | 1 | ? | 1 | 1 | 1 | 0 | 5 |
| Qutishat 2021 | 1 | 0 | 1 | 1 | ? | 1 | 1 | 1 | ? | 6 |
| Reid 2022 | 1 | 1 | ? | 1 | 1 | 1 | 1 | 1 | 1 | 8 |
| Renzi 2023 | 1 | 0 | ? | 1 | ? | 1 | 1 | 1 | ? | 5 |
| Roberts 2021 | 1 | 1 | ? | 1 | 1 | 1 | 1 | 1 | 1 | 8 |
| Sar-El 2022 | 1 | 1 | ? | 1 | ? | 1 | 1 | ? | 1 | 6 |
| Seifeldin Abdeen 2023 | 1 | 0 | ? | 1 | ? | 1 | 1 | 1 | ? | 5 |
| Shechter 2022 | 1 | 1 | ? | 1 | ? | 1 | 1 | 1 | 0 | 6 |
| Sobregrau Sangra 2022 | 1 | 1 | ? | 1 | ? | 1 | ? | 1 | 0 | 5 |
| Somi 2022 | 1 | 1 | 1 | 0 | ? | 1 | 1 | 1 | 0 | 6 |
| Song 2020 | 1 | 0 | ? | 1 | ? | 1 | 1 | 1 | ? | 5 |
| Stafseth 2022 | 1 | 1 | ? | 1 | ? | 1 | 1 | 1 | 0 | 6 |
| Stanislawski 2023 | 1 | 1 | ? | 1 | ? | 1 | 1 | 1 | 0 | 6 |
| Styra 2021 | 1 | 1 | 1 | 1 | ? | 1 | 1 | 1 | 0 | 7 |
| Tatsuno 2021 | 1 | 0 | 1 | 1 | ? | 1 | 1 | 1 | ? | 6 |
| Villalba-Arias 2021 | 1 | 1 | ? | 1 | ? | 1 | 1 | 1 | 1 | 7 |
| Wang 2020 | 1 | 1 | 1 | 1 | ? | 1 | 1 | 1 | 0 | 7 |
| Wanigasooriya 2021 | 1 | 1 | ? | 1 | 1 | 1 | 1 | 1 | 0 | 7 |
| Wild 2022 | 1 | 1 | ? | 1 | ? | 1 | 1 | 1 | 0 | 6 |
| Wojcik 2022 | 1 | 1 | ? | 1 | ? | 1 | 1 | 1 | 0 | 6 |
| Yalcin 2020 | 1 | 1 | ? | 1 | ? | 1 | 1 | 1 | 0 | 6 |
| Yang 2022 | 1 | 1 | 1 | 1 | ? | 1 | 1 | 1 | 1 | 8 |
| Yeo 2021 | 1 | 1 | ? | 1 | ? | 1 | 1 | 1 | 1 | 7 |
| Yin 2020 | 1 | 0 | ? | 1 | ? | 1 | 1 | 1 | ? | 5 |
| Yin 2021 | 1 | 1 | ? | 1 | ? | 1 | 1 | 1 | 1 | 7 |
| Zakeri 2021 | 1 | 1 | ? | 1 | ? | 1 | ? | 1 | 1 | 6 |
| Zhang 2020 | 1 | 0 | ? | 1 | ? | 1 | 1 | 1 | 0 | 5 |
| Zhang 2021 | 1 | 0 | ? | 1 | ? | 1 | 1 | 1 | ? | 5 |
| Zhang 2022a | 1 | ? | ? | 1 | ? | 1 | ? | 1 | 1 | 5 |
| Zhang 2022b | 1 | 1 | ? | 1 | 1 | 1 | 1 | 1 | 1 | 8 |

Table 3. Quality assessment scores for each paper included in the review, based on the JBI Critical Appraisal Checklist for Studies Reporting Prevalence Data.

Abbreviations: Q1. Was the sample frame appropriate to address the target population?

Q2. Were study participants recruited in an appropriate way?

Q3. Was the sample size adequate?

Q4. Were the study subjects and setting described in detail?

Q5. Was data analysis conducted with sufficient coverage of the identified sample?

Q6. Were valid methods used for the identification of the condition?

Q7. Was the condition measured in a standard, reliable way for all participants?

Q8. Was there appropriate statistical analysis?

Q9. Was the response rate adequate, if not, was response rate managed appropriately?

QA, Quality Assessment.

1, Yes; 0, No; ?, Unclear.

# Figure 1. Pooled prevalence estimates across PTSD screening tools

Figure 1. Estimated pooled prevalence of probable PTSD for studies using various types of screening tools, both prior to and during COVID-19. IES comprises IES-R and abbreviated versions. PCL comprises PCL-C, PCL-5 and abbreviated versions. PC-PTSD comprises both PC-PTSD and PC-PTSD-5. Other comprises TSQ, ITQ, DTS, SCID-5, PDS-5, PTSS-10 and PTSS-14.

Abbreviations: DTS, Davidson Trauma Scale; DTS-8, 8-item DTS; IES-R, Impact of Event Scale – Revised; ITQ, International Trauma Questionnaire; PCL, PTSD Checklist; PCL-5, PCL for DSM-5; PCL-C, PCL-Civilian; PC-PTSD, Primary Care-PTSD Screen; PC-PTSD-5, PC-PTSD for DSM-5; PDS-5, Post-traumatic Diagnostic Scale for DSM-5; PTSD, Post-traumatic stress disorder; PTSS-10, 10-item Post-traumatic Stress Scale; PTSS-14, 14-item Post-traumatic Stress Scale; SCID-5, Structural Clinical Interview for DSM-5 Disorders; TSQ, Trauma Screening Questionnaire.

|  | \| **Number of studies** \| \| --- \| |  | **Prevalence** | **Lower Limit** | **Upper Limit** | **P-value** | **I^2^** |
| --- | --- | --- | --- | --- | --- | --- | --- | --- |
| **Prior to COVID-19** | |  |  |  |  | 0.047 |  |
| IES | 5 |  | 21.02% | 17.36% | 25.22% |  | 44.55 |
| PCL | 7 |  | 15.38% | 10.71% | 21.59% |  | 92.02 |
| PC-PTSD | 3 |  | 22.37% | 14.56% | 32.78% |  | 97.11 |
| Other | 6 |  | 10.20% | 5.63% | 17.80% |  | 96.96 |
| **During COVID-19** | |  |  |  |  | 0.016 |  |
| IES | 48 |  | 28.69% | 25.45% | 32.16% |  | 98.05 |
| PCL | 56 |  | 20.03% | 16.27% | 24.41% |  | 99.12 |
| PC-PTSD | 12 |  | 28.05% | 19.70% | 38.26% |  | 98.03 |
| Other | 13 |  | 29.43% | 22.56% | 37.38% |  | 96.75 |
|  |  |  |  |  |  |  |  |
|  |  |  |  |  |  |  |  |

# Figure 2. Pooled prevalence estimates for low, fair and high quality studies prior to and during COVID-19

|  | \|  \| \| --- \| |  |  |  |  |  |  |
| --- | --- | --- | --- | --- | --- | --- | --- | --- |
|  | **Number of studies** | **Estimated pooled prevalence of probable PTSD** | **Prevalence** | **Lower Limit** | **Upper Limit** | **P-value** | **I^2^** |
| **Prior to COVID-19** | |  |  |  |  | p<0.001 |  |
| High quality | 5 |  | 17.1% | 14.1% | 19.7% |  | 80.5 |
| Fair quality | 15 |  | 14.1% | 9.9% | 20.5% |  | 96.7 |
| Low quality | 1 |  | 24.5% | 21.6% | 27.6% |  | NA |
| **During COVID-19** | |  |  |  |  | p=0.017 |  |
| High quality | 42 |  | 19.8% | 15.6% | 24.9% |  | 99.3 |
| Fair quality | 81 |  | 27.5% | 24.2% | 31.0% |  | 98.7 |
| Low quality | 4 |  | 30.9% | 24.2% | 38.7% |  | 78.6 |
|  |  |  |  |  |  |  |  |
|  |  |  |  |  |  |  |  |

Figure 2. Estimated pooled prevalence of probable PTSD based on studies that were considered high-quality, fair quality, and low-quality both prior to and during COVID-19. Abbreviations: PTSD denotes post-traumatic stress disorder

# Figure 3. Pooled prevalence estimates among studies with an adequate sampling strategy and adequate response rate vs those without

Figure 3. Estimated pooled prevalence of probable PTSD for studies with an adequate sampling strategy and adequate response rate vs studies with inadequate sampling strategy and/or response rate, both prior to and during COVID-19. Abbreviations: PTSD denotes post-traumatic stress disorder

|  | \| **Number of studies** \| \| --- \| |  | **Prevalence** | **Lower Limit** | **Upper Limit** | **P-value** | **I^2^** |
| --- | --- | --- | --- | --- | --- | --- | --- | --- |
| **Prior to COVID-19** |  |  |  |  |  | 0.57 |  |
| Adequate sampling and response rate | 5 |  | 14.26% | 10.16% | 19.65% |  | 90.0 |
| Inadequate sampling and/or response rate | 16 |  | 16.17% | 12.08% | 21.32% |  | 96.5 |
| **During COVID-19** |  |  |  |  |  | 0.11 |  |
| Adequate sampling and response rate | 35 |  | 20.64% | 15.23% | 27.36% |  | 99.5 |
| Inadequate sampling and/or response rate | 92 |  | 26.55% | 23.74% | 29.57% |  | 98.4 |
|  |  |  |  |  |  |  |  |
|  |  |  |  |  |  |  |  |

# Figure 4. Meta-regression of prevalence over year prior to COVID-19

**
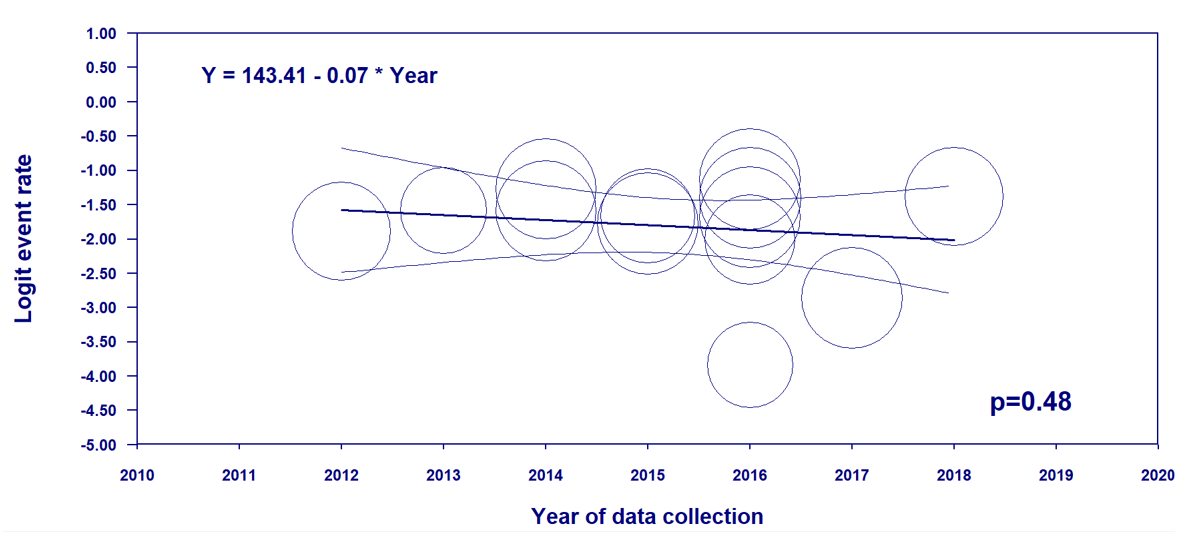
**

Figure 4. Scatterplot demonstrating logit event rate over year of data collection for all studies prior to COVID-19. Regression line with 95%CI. β = -0.07 (95%CI: -0.27 to 0.13), SE = 0.10, p=0.48, I^2^ = 96.2%.

# Figure 5a. One-Study Removed Forest Plot for Studies Prior to COVID-19

Figure 5a. Forest plot demonstrating probable PTSD prevalence estimates using the one-study removed sensitivity analysis for studies prior to COVID-19. Abbreviations: CI, Confidence Intervals

# Figure 5b. One-Study Removed Forest Plot for Studies During COVID-19

Figure 5b. Forest plot demonstrating probable PTSD prevalence estimates using the one-study removed sensitivity analysis for studies during COVID-19. Abbreviations: CI, Confidence Intervals

# Figure 6. Funnel Plots Visualizing Possible Publication Bias

**A**

**-4**

**-3**

**-2**

**-1**

**0**

**1**

**2**

**3**

**4**

**0.0**

**0.2**

**0.4**

**0.6**

**0.8**

**Standard Error**

**Logit event rate**

**-5**

**-4**

**-3**

**-2**

**-1**

**0**

**1**

**2**

**3**

**4**

**5**

**0.0**

**0.5**

**1.0**

**1.5**

**2.0**

**Logit event rate**

Figure 6. Funnel plots examining possible publication bias (A) prior to COVID-19 and (B) during COVID-19

**B**

# References

1. Davidson JR, Book S, Colket J, Tupler L, Roth S, David D, et al. Assessment of a new self-rating scale for post-traumatic stress disorder. Psychol Med. 1997;27(1):153-60.

2. Connor KM, Davidson JR. Further psychometric assessment of the TOP‐8: A brief interview‐based measure of PTSD. Depress Anxiety. 1999;9(3):135-7.

3. Morina N, Ehring T, Priebe S. Diagnostic utility of the impact of event scale–revised in two samples of survivors of war. PloS One. 2013;8(12):e83916.

4. Creamer M, Bell R, Failla S. Psychometric properties of the impact of event scale—revised. Behav Res Ther. 2003;41(12):1489-96.

5. Hosey MM, Leoutsakos J-MS, Li X, Dinglas VD, Bienvenu OJ, Parker AM, et al. Screening for posttraumatic stress disorder in ARDS survivors: validation of the Impact of Event Scale-6 (IES-6). Crit Care. 2019;23:1-7.

6. Hansen M, Vægter HB, Cloitre M, Andersen TE. Validation of the Danish International Trauma Questionnaire for posttraumatic stress disorder in chronic pain patients using clinician-rated diagnostic interviews. Eur J Psychotraumato. 2021;12(1):1880747.

7. Blanchard EB, Jones-Alexander J, Buckley TC, Forneris CA. Psychometric properties of the PTSD Checklist (PCL). Behav Res Ther. 1996;34(8):669-73.

8. Bovin MJ, Marx BP, Weathers FW, Gallagher MW, Rodriguez P, Schnurr PP, et al. Psychometric properties of the PTSD checklist for diagnostic and statistical manual of mental disorders–fifth edition (PCL-5) in veterans. Psychol Assess. 2016;28(11):1379.

9. Geier TJ, Hunt JC, Hanson JL, Heyrman K, Larsen SE, Brasel KJ, et al. Validation of abbreviated four‐and eight‐item versions of the PTSD checklist for DSM‐5 in a traumatically injured sample. J Trauma Stress. 2020;33(3):218-26.

10. Prins A, Ouimette P, Kimerling R, R.P C, Hugelshofer DS, al. e. The primary care PTSD screen (PC-PTSD): development and operating characteristics. Prim Care Psychiatry. 2003;9(1):9-14.

11. Prins A, Bovin MJ, Smolenski DJ, Marx BP, Kimerling R, Jenkins-Guarnieri MA, et al. The primary care PTSD screen for DSM-5 (PC-PTSD-5): development and evaluation within a veteran primary care sample. J Gen Intern Med. 2016;31(10):1206-11.

12. Foa EB, McLean CP, Zang Y, Zhong J, Powers MB, Kauffman BY, et al. Psychometric properties of the Posttraumatic Diagnostic Scale for DSM–5 (PDS–5). Psychol Assess. 2016;28(10):1166.

13. Twigg E, Humphris G, Jones C, Bramwell R, Griffiths RD. Use of a screening questionnaire for post‐traumatic stress disorder (PTSD) on a sample of UK ICU patients. Acta Anaesthesiol. Scand. 2008;52(2):202-8.

14. Hansen M, Andersen TE, Armour C, Elklit A, Palic S, Mackrill T. PTSD-8: a short PTSD inventory. Clin Pract Epidemiol Ment Health. 2010;6:101.

15. Lang AJ, Stein MB. An abbreviated PTSD checklist for use as a screening instrument in primary care. Behav Res Ther. 2005;43(5):585-94.

16. Sin GL, Abdin E, Lee J. The PSS‐SR as a screening tool for PTSD in first‐episode psychosis patients. Early Interv Psychiatry. 2012;6(2):191-4.

17. Brewin CR, Rose S, Andrews B, Green J, Tata P, McEvedy C, et al. Brief screening instrument for post-traumatic stress disorder. Br J Psychiatry. 2002;181(2):158-62.

18. Breslau N, Peterson EL, Kessler RC, Schultz LR. Short screening scale for DSM-IV posttraumatic stress disorder. Am J Psychaitry. 1999;156(6):908-11.
